# Supplementary material for: Reciprocal causation mixture model for robust Mendelian randomization analysis using genome-scale summary data
Source: Nat Commun. 2023 Feb 28;14:1131. doi: 10.1038/s41467-023-36490-4 (PMC9975185; doi:10.1038/s41467-023-36490-4)
Supplement: Supplementary file 1 — Supplementary information [file 41467_2023_36490_MOESM1_ESM.pdf]

Supplementary Information

Supplementary Note
Model definition
For a pair of reciprocally caused phenotypes (Y_1 and Y_2), SNPs are classified into four mutually exclusive components:

- Y_1-specific component (G_1): SNPs that contribute directly to Y_1 only;
- Y_2-specific component (G_2): SNPs that contribute directly to Y_2 only;
- pleiotropic component (G_C): SNPs that contribute directly to both phenotypes;
- null component (G_0): SNPs with no direct effects on either phenotype.

The proportions of all SNPs in the four components are π_1, π_2, π_c and π_0.

The two phenotypes can be written in the form:

$$Y_1 = \delta_{12} Y_2 + \sum_{i \in G_1} \gamma_{1i} X_i + \sum_{l \in G_C} \gamma_{C1l} X_l + e_1$$

$$Y_2 = \delta_{21} Y_1 + \sum_{j \in G_2} \gamma_{2j} X_j + \sum_{l \in G_C} \gamma_{C2l} X_l + e_2$$

where X_i, X_j and X_l represent standardized Y_1-specific, Y_2-specific and pleiotropic SNP genotypes, respectively; $\gamma_{1i}, \gamma_{2j}, \gamma_{C1l}, \gamma_{C2l}$ denote the direct effect sizes of phenotype-specific and pleiotropic SNPs for Y_1 and Y_2 with $i \in G_1, j \in G_2, l \in G_C$; δ_{12} is the casual effect of $Y_2 \to Y_1$ and δ_{21} is the causal effect of $Y_1 \to Y_2$; e_1 and e_2 are the residual effects. We could convert the above formula into the following matrix form:

$$\boldsymbol{Y} = [\boldsymbol{I} - \boldsymbol{\Delta}]^{-1}\left[\boldsymbol{\Gamma}^{(G_1)}\boldsymbol{X}^{(G_1)} + \boldsymbol{\Gamma}^{(G_2)}\boldsymbol{X}^{(G_2)} + \boldsymbol{\Gamma}^{(G_C)}\boldsymbol{X}^{(G_C)} + \boldsymbol{e}\right]$$

where, $[\boldsymbol{I} - \boldsymbol{\Delta}]^{-1} = \frac{1}{1-\delta_{12}\delta_{21}}\begin{bmatrix} 1 & \delta_{12} \\ \delta_{21} & 1 \end{bmatrix}$, $\boldsymbol{X}^{(h)}$ and $\boldsymbol{\Gamma}^{(h)}$ represent the standardized genotype and the direct effect of SNPs in component h with $h \in (G_0, G_1, G_2, G_C)$. The null SNP component is not included in the formula.

In our model, we assume the direct causal effects follow the distribution:

$$\gamma_{1i} \sim N(0, \sigma_1^2) \quad (1)$$

$$\gamma_{2j} \sim N(0, \sigma_2^2) \quad (2)$$

$$\begin{pmatrix} \gamma_{C1l} \\ \gamma_{C2l} \end{pmatrix} \sim N\left[\begin{pmatrix} 0 \\ 0 \end{pmatrix}, \begin{pmatrix} \sigma_{C1}^2 & \rho_{C1,C2} \\ \rho_{C1,C2} & \sigma_{C2}^2 \end{pmatrix}\right] \quad (3)$$

Here, σ_1^2 and σ_2^2 denote the per-SNP variance of G_1 and G_2, and σ_{C1}^2 and σ_{C2}^2 are the per-SNP variance of G_C for Y_1 and Y_2, respectively with a covariance of $\rho_{C1,C2}$.

Thus, under the reciprocal joint model, a pair of phenotypes (as a sum of individual contributions from genetic variants) can be described as a joint linear model: $\boldsymbol{Y} = [\boldsymbol{I} - \boldsymbol{\Delta}]^{-1} \sum_{k=1}^{K} \boldsymbol{\Gamma}_k^{(h)} X_k + \boldsymbol{\varepsilon}$, where $\boldsymbol{\Gamma}_k^{(h)}$ is the direct effect of the k-th SNP on the phenotypes depending on its component membership h, and X_k is the standardized genotype for the k-th SNP, $\boldsymbol{\varepsilon}$ is the residual effect.

Mixture form of the marginal estimate

For univariate phenotype, according to linear relationship between the marginal and joint regression coefficients [1,2], $\tau_k = \sum_{i=1}^{N_k^*} \beta_i \rho_{ki}$, where τ_k denotes the marginal effect size for the k-th SNP, N_k^* is the total number of SNPs tagged by the k-th SNP, ρ_{ki} is the LD correlation between k-th and i-th SNP, β_i is the joint effect size of the i-th SNP tagged by the k-th SNP. Thus, the summary-level estimation $\hat{\tau}_k$ can be divided into a mixture form:

$$\hat{\tau}_k = \sum_{i=1}^{N_k^*} \beta_i \rho_{ki} + \varepsilon = \sum_{i=1}^{N_k^{(G_1)}} \beta_i^{(G_1)} \rho_{ki} + \sum_{i=1}^{N_k^{(G_2)}} \beta_i^{(G_2)} \rho_{ki} + \sum_{i=1}^{N_k^{(G_C)}} \beta_i^{(G_C)} \rho_{ki} + \sum_{i=1}^{N_k^{(G_0)}} \beta_i^{(G_0)} \rho_{ki} + \varepsilon \tag{4}$$

where $\beta_i^{(h)}$ is the joint effect size for the i-th SNP belonging to component h; $N_k^{(h)}$ is a latent variable denoting the number of h-component SNPs tagged by the k-th SNP, and $N_k^* = N_k^{(G_1)} + N_k^{(G_2)} + N_k^{(G_C)} + N_k^{(G_0)}$.

Composite likelihood in the mixture form

Under our reciprocal joint model for two phenotypes, we assumed a bivariate normal distribution of the marginal estimate for the k-th SNP from the GWAS summary statistics:

$$\hat{\boldsymbol{\tau}}_{\boldsymbol{k}} = \begin{pmatrix} \hat{\tau}_{1k} \\ \hat{\tau}_{2k} \end{pmatrix} \sim \sum_{\mathbb{N}_k} \Pr(\mathbb{N}_k) N \left[\begin{pmatrix} 0 \\ 0 \end{pmatrix}, \begin{pmatrix} \sigma_{\hat{\tau}_{1k}}^2 & \rho_{\hat{\tau}_{1k},\hat{\tau}_{2k}} \\ \rho_{\hat{\tau}_{1k},\hat{\tau}_{2k}} & \sigma_{\hat{\tau}_{2k}}^2 \end{pmatrix} \right] \tag{5}$$

Here, $\hat{\tau}_{1k}$ and $\hat{\tau}_{2k}$ represent the marginal estimates of the k-th SNP from GWAS summary statistics of phenotype Y_1 and Y_2 respectively. $\mathbb{N}_k$ is a random vector of $\left(N_k^{(G_1)}, N_k^{(G_2)}, N_k^{(G_C)}, N_k^{(G_0)}\right)$. According to the multinomial distribution with total counts $N_k^* = N_k^{(G_1)} + N_k^{(G_2)} + N_k^{(G_C)} + N_k^{(G_0)}$ and cell probabilities $(\pi_1, \pi_2, \pi_C, \pi_0)$, we can calculate the probability distribution of $\mathbb{N}_k$: $\Pr(\mathbb{N}_k) = \frac{N_k^*!}{N_k^{(G_1)}! N_k^{(G_2)}! N_k^{(G_C)}! N_k^{(G_0)}!} (\pi_1)^{N_k^{(G_1)}} (\pi_2)^{N_k^{(G_2)}} (\pi_C)^{N_k^{(G_C)}} (\pi_0)^{N_k^{(G_0)}}$, where $\pi_1, \pi_2, \pi_C, \pi_0$ represent the mixing proportion of the corresponding component. $\begin{pmatrix} \sigma_{\hat{\tau}_{k1}}^2 & \rho_{\hat{\tau}_{k1},\hat{\tau}_{k2}} \\ \rho_{\hat{\tau}_{k1},\hat{\tau}_{k2}} & \sigma_{\hat{\tau}_{k2}}^2 \end{pmatrix}$ is the variance-covariance matrix for $(\hat{\tau}_{1k}, \hat{\tau}_{2k})$.

We define $\boldsymbol{\beta}_k^{(h)} = \begin{pmatrix} \beta_{1k}^{(h)} \\ \beta_{2k}^{(h)} \end{pmatrix} = [\boldsymbol{I} - \boldsymbol{\Delta}]^{-1} \boldsymbol{\Gamma}_k^{(h)}$ as the joint effect size depending on the component condition h of the k-th SNP, where $\beta_{1k}^{(h)}, \beta_{2k}^{(h)}$ are the joint effect sizes of the k-th SNP on phenotype Y_1, Y_2 respectively. According to the direct effect size distribution (1), (2) and (3), we can derive the component-dependent variance-covariance matrix of $(\beta_{1k}^{(h)}, \beta_{2k}^{(h)})$:

$$\Sigma_{(G_1)} = \begin{bmatrix} var\left(\beta_{1k}^{(G_1)}\right) & cov\left(\beta_{1k}^{(G_1)}, \beta_{2k}^{(G_1)}\right) \\ cov\left(\beta_{1k}^{(G_1)}, \beta_{2k}^{(G_1)}\right) & var\left(\beta_{2k}^{(G_1)}\right) \end{bmatrix} = \begin{bmatrix} \frac{\sigma_1^2}{(1-\delta_{12}\delta_{21})^2} & \frac{\delta_{21}\sigma_1^2}{(1-\delta_{12}\delta_{21})^2} \\ \frac{\delta_{21}\sigma_1^2}{(1-\delta_{12}\delta_{21})^2} & \frac{\delta_{21}^2\sigma_1^2}{(1-\delta_{12}\delta_{21})^2} \end{bmatrix} \tag{6}$$

$$\Sigma_{(G_2)} = \begin{bmatrix} var\left(\beta_{1k}^{(G_2)}\right) & cov\left(\beta_{1k}^{(G_2)}, \beta_{2k}^{(G_2)}\right) \\ cov\left(\beta_{1k}^{(G_2)}, \beta_{2k}^{(G_2)}\right) & var\left(\beta_{2k}^{(G_2)}\right) \end{bmatrix} = \begin{bmatrix} \frac{\delta_{12}^2\sigma_2^2}{(1-\delta_{12}\delta_{21})^2} & \frac{\delta_{12}\sigma_2^2}{(1-\delta_{12}\delta_{21})^2} \\ \frac{\delta_{12}\sigma_2^2}{(1-\delta_{12}\delta_{21})^2} & \frac{\sigma_2^2}{(1-\delta_{12}\delta_{21})^2} \end{bmatrix} \tag{7}$$

$$\Sigma_{(G_C)} = \begin{bmatrix} var\left(\beta_{1k}^{(G_C)}\right) & cov\left(\beta_{1k}^{(G_C)}, \beta_{2k}^{(G_C)}\right) \\ cov\left(\beta_{1k}^{(G_C)}, \beta_{2k}^{(G_C)}\right) & var\left(\beta_{2k}^{(G_C)}\right) \end{bmatrix} = \begin{bmatrix} \frac{\sigma_{C1}^2 + \delta_{12}^2\sigma_{C2}^2 + 2\delta_{12}\rho_{C1,C2}}{(1-\delta_{12}\delta_{21})^2} & \frac{\delta_{21}\sigma_{C1}^2 + \delta_{12}\sigma_{C2}^2 + (1+\delta_{12}\delta_{21})\rho_{C1,C2}}{(1-\delta_{12}\delta_{21})^2} \\ \frac{\delta_{21}\sigma_{C1}^2 + \delta_{12}\sigma_{C2}^2 + (1+\delta_{12}\delta_{21})\rho_{C1,C2}}{(1-\delta_{12}\delta_{21})^2} & \frac{\sigma_{C2}^2 + \delta_{21}^2\sigma_{C1}^2 + 2\delta_{21}\rho_{C1,C2}}{(1-\delta_{12}\delta_{21})^2} \end{bmatrix} \tag{8}$$

Based on the definition of LD-score, we could obtain the component-dependent LD-score for the k-th SNP in equation (4) in the form: $\ell_k^{(h)} = \sum_{i=1}^{N_k^{(h)}} \rho_{ki}^2$. In practice it is not feasible to consider all possible combinations of components of tagged SNPs to calculate this $\ell_k^{(h)}$. However, under the assumption that LD patterns are independent of the probability of SNP-effects belonging to different mixture components, we could follow the approximation from Zhang *et al*[2] as:

$$\ell_k^{(h)} = \sum_{i=1}^{N_k^{(h)}} \rho_{ki}^2 \approx \frac{N_k^{(h)}}{N_k^*} \sum_{i=1}^{N_k^*} \rho_{ki}^2 = \frac{N_k^{(h)}}{N_k^*} \ell_k \quad (9)$$

where ℓ_k is the LD-score for the k-th SNP and can be substituted with LD-score data from a reference genome (e.g. 1000 Genomes Project).

Thus, from equations (4), (6), (7), (8) and (9), we can derive the variance-covariance matrix for $(\hat{\tau}_{1k}, \hat{\tau}_{2k})$:

$$\sigma_{\hat{\tau}_{1k}}^2 \approx \frac{\sigma_1^2}{(1-\delta_{12}\delta_{21})^2} \frac{N_k^{(G_1)}}{N_k^*} \ell_k + \frac{\delta_{12}^2 \sigma_2^2}{(1-\delta_{12}\delta_{21})^2} \frac{N_k^{(G_2)}}{N_k^*} \ell_k + \frac{[\sigma_{C1}^2 + \delta_{12}^2 \sigma_{C2}^2 + 2\delta_{12}\rho_{C1,C2})]}{(1-\delta_{12}\delta_{21})^2} \frac{N_k^{(G_C)}}{N_k^*} \ell_k + a_1 + 1/n_1$$

$$\sigma_{\hat{\tau}_{2k}}^2 \approx \frac{\delta_{21}^2 \sigma_1^2}{(1-\delta_{12}\delta_{21})^2} \frac{N_k^{(G_1)}}{N_k^*} \ell_k + \frac{\sigma_2^2}{(1-\delta_{12}\delta_{21})^2} \frac{N_k^{(G_2)}}{N_k^*} \ell_k + \frac{[\sigma_{C2}^2 + \delta_{21}^2 \sigma_{C1}^2 + 2\delta_{21}\rho_{C1,C2}]}{(1-\delta_{12}\delta_{21})^2} \frac{N_k^{(G_C)}}{N_k^*} \ell_k + a_2 + 1/n_2$$

$$\rho_{\hat{\tau}_{1k},\hat{\tau}_{2k}} \approx \frac{\delta_{21}\sigma_1^2}{(1-\delta_{12}\delta_{21})^2} \frac{N_k^{(G_1)}}{N_k^*} \ell_k + \frac{\delta_{12}\sigma_2^2}{(1-\delta_{12}\delta_{21})^2} \frac{N_k^{(G_2)}}{N_k^*} \ell_k + \frac{[\delta_{21}\sigma_{C1}^2 + \delta_{12}\sigma_{C2}^2 + (1+\delta_{12}\delta_{21})\rho_{C1,C2}]}{(1-\delta_{12}\delta_{21})^2} \frac{N_k^{(G_C)}}{N_k^*} \ell_k + \rho_0$$

where n_1 and n_2 are the sample size for the two GWAS; a_1 and a_2 are additional inflation factors accounting for systematic bias in variance estimates for phenotype Y_1 and Y_2 respectively; ρ_0 is a factor accounting for bias in the covariance estimates due to effects such as sample overlapping.

Then, the likelihood for the summary-statistic of the k-th SNP is: $L(\boldsymbol{\theta}; \hat{\boldsymbol{\tau}}_{\boldsymbol{k}}) = p(\hat{\boldsymbol{\tau}}_{\boldsymbol{k}} | \boldsymbol{\theta}) = \sum_{\mathbb{N}_k} \Pr(\mathbb{N}_k) f(\hat{\tau}_{1k}, \hat{\tau}_{1k})$, where $f(\hat{\tau}_{1k}, \hat{\tau}_{2k})$ is the density function of bivariate normal distribution with $\boldsymbol{\theta} = (\pi_1, \pi_2, \pi_c, \sigma_1^2, \sigma_2^2, \sigma_{C1}^2, \sigma_{C2}^2, \rho_{C1,C2}, \delta_{12}, \delta_{21}, a_1, a_2, \rho_0)$.

Thus, the composite log-likelihood function is in the form:

$$CL(\boldsymbol{\theta}; \hat{\boldsymbol{\tau}}_{\boldsymbol{k}}) = \sum_{k=1}^{K} log L(\boldsymbol{\theta}; \hat{\boldsymbol{\tau}}_{\boldsymbol{k}}) = \sum_{k=1}^{K} log \left[\sum_{\mathbb{N}_k} \Pr(\mathbb{N}_k) f(\hat{\tau}_{1k}, \hat{\tau}_{2k}) \right] \quad (10)$$

So, the maximum composite likelihood estimator can be given by

$$\widehat{\boldsymbol{\theta}} = \underset{\boldsymbol{\theta}}{argmax}\, CL(\boldsymbol{\theta}; \hat{\boldsymbol{\tau}}_{\boldsymbol{k}})$$

Implementation

We estimate the parameters from equation (10) using an Expectation-Maximization algorithm. In E-step, under the current parameter estimate $\boldsymbol{\theta}^{(t)}$:

$$Q(\boldsymbol{\theta}|\boldsymbol{\theta}^{(t)}) = E_{\mathbb{N}_k|\hat{\boldsymbol{\tau}},\boldsymbol{\theta}^{(t)}}\{CL(\boldsymbol{\theta}; \hat{\boldsymbol{\tau}})\} = \sum_{k=1}^{K} \sum_{\mathbb{N}_k} \Pr_{(t)}(\mathbb{N}_k | \hat{\boldsymbol{\tau}}_{\boldsymbol{k}}, \boldsymbol{\theta}^{(t)}) log[\Pr(\mathbb{N}_k) f(\hat{\tau}_{1k}, \hat{\tau}_{2k})]$$

where, $f(\hat{\tau}_{1k}, \hat{\tau}_{2k})$ is the density function of bivariate normal distribution and $\Pr_{(t)}(\mathbb{N}_k|\hat{\boldsymbol{\tau}}_k, \boldsymbol{\theta}^{(t)}) = \frac{\Pr_{(t)}(\mathbb{N}_k) f(\hat{\tau}_{1k}, \hat{\tau}_{2k})}{\sum_{\mathbb{N}_k} \Pr_{(t)}(\mathbb{N}_k) f(\hat{\tau}_{1k}, \hat{\tau}_{2k})}$. In M-step, parameters for mixing proportions (π_1, π_2 and π_c) have a closed form:

$$\pi_1^{(t+1)} = \frac{(1 - \pi_2^{(t)} - \pi_C^{(t)}) \sum_{k=1}^{K} \sum_{\mathbb{N}_k} \Pr_{(t)}(\mathbb{N}_k|\hat{\boldsymbol{\tau}}_k, \boldsymbol{\theta}^{(t)}) N_k^{(G_1)}}{\sum_{k=1}^{K} \sum_{\mathbb{N}_k} \Pr_{(t)}(\mathbb{N}_k|\hat{\boldsymbol{\tau}}_k, \boldsymbol{\theta}^{(t)}) (N_k^{(G_0)} + N_k^{(G_1)})}$$

$$\pi_2^{(t+1)} = \frac{(1 - \pi_1^{(t)} - \pi_C^{(t)}) \sum_{k=1}^{K} \sum_{\mathbb{N}_k} \Pr_{(t)}(\mathbb{N}_k|\hat{\boldsymbol{\tau}}_k, \boldsymbol{\theta}^{(t)}) N_k^{(G_2)}}{\sum_{k=1}^{K} \sum_{\mathbb{N}_k} \Pr_{(t)}(\mathbb{N}_k|\hat{\boldsymbol{\tau}}_k, \boldsymbol{\theta}^{(t)}) (N_k^{(G_0)} + N_k^{(G_2)})}$$

$$\pi_C^{(t+1)} = \frac{(1 - \pi_1^{(t)} - \pi_2^{(t)}) \sum_{k=1}^{K} \sum_{\mathbb{N}_k} \Pr_{(t)}(\mathbb{N}_k|\hat{\boldsymbol{\tau}}_k, \boldsymbol{\theta}^{(t)}) N_k^{(G_C)}}{\sum_{k=1}^{K} \sum_{\mathbb{N}_k} \Pr_{(t)}(\mathbb{N}_k|\hat{\boldsymbol{\tau}}_k, \boldsymbol{\theta}^{(t)}) (N_k^{(G_0)} + N_k^{(G_C)})}$$

It is difficult to derive the close form for parameters of effect size variances ($\sigma_1^2, \sigma_2^2, \sigma_{C1}^2, \sigma_{C2}^2$ and $\rho_{C1,C2}$) and the reciprocal causation (δ_{12} and δ_{21}), thus they were estimated by Nelder-Mead optimization.

To improve the efficiency of our algorithm, we made several further adaptions. The details are as follows:

i. It is reasonable to assume a small number of true causal SNPs tagged by the k-th SNP. Thus, in practice, we set a constraint $N_k^{(h)} \leq 3$ with $h \in (G_1, G_2, G_C)$. We have tested this setting in both simulation and real data and found it could substantially decrease the computation burden while keeping reasonable genetic effect estimates.
ii. The variance-covariance matrix $\begin{pmatrix} \sigma_{C1}^2 & \rho_{C1,C2} \\ \rho_{C1,C2} & \sigma_{C2}^2 \end{pmatrix}$ should always be positive-definite;
iii. To ensure the convergence of reciprocal causation between the two phenotypes, $|\delta_{12}|$ and $|\delta_{21}|$ should each be less than 1.0.
iv. For variance calculation, it is arduous to obtain the derivatives for parameters directly from the composite likelihood function. Thus, we took symmetric derivatives to efficiently calculate derivatives required. The first-order partial derivative with respect to x is $\frac{\partial f}{\partial x} = \lim_{h \to 0} \frac{f(x+h) - f(x-h)}{2h}$; the second-order derivative with respect to x is $\frac{\partial^2 f}{\partial x^2} = \lim_{h \to 0} \frac{f(x+h) - 2f(x) + f(x-h)}{h^2}$ and the second-order mixed derivative is $\frac{\partial^2 f}{\partial x \partial y} = \lim_{h \to 0} \frac{[f(x+h, y+h) - f(x-h, y+h)] - [f(x+h, y-h) - f(x+h, y-h)]}{4h^2}$. Here, f is the objective function and x, y denote the corresponding parameters.

Calculation of initial weight for each model

We optimized the weights of each model under the full-model likelihood function. The initial weight for each model was calculated based on a modified Akaike information criterion (AIC) for composite likelihood[3]. AIC of the s-th model (AIC_s) can be written as:

$$AIC_s = -2CL(\widehat{\boldsymbol{\theta}}_s; \hat{\boldsymbol{\tau}}) + 2d_s$$

$\widehat{\boldsymbol{\theta}}_s$ are parameter estimates in the s-th model and $d_s = tr(I(\boldsymbol{\theta}_s)^{-1} J(\boldsymbol{\theta}_s))$. Here, $I(\boldsymbol{\theta}_s)$ and $J(\boldsymbol{\theta}_s)$ can be estimated by plugging in the estimated parameter values $\widehat{\boldsymbol{\theta}}_s$ as previously described. Then, the weight for the s-th model is defined[4 5] as

$$\widehat{w}_s = \frac{\exp(0.5\Delta AIC_s)}{\sum_s^S \exp(0.5\Delta AIC_s)}$$

where ΔAIC_s is the normalized AIC for the s-th model by $AIC_s - max_{s \in \boldsymbol{S}} AIC_s$. In this way, the weights could sum up to one by definition.

Effect size transformation for binary phenotypes

When the phenotype is binary, the estimates of the reciprocal causal path are on the liability scale, thus for binary phenotypes we have to convert the summary-level odds ratio (OR) to the equivalent effect size estimation on the liability scale. To this aim, we first used minor allele frequency (f) to adjust the reported $ln\widehat{OR}$ to the standardized form $\left(ln\widehat{OR}\right)_{std} = \sqrt{2f(1-f)} \times ln\widehat{OR}$, and $var\left[\left(ln\widehat{OR}\right)_{std}\right] = 2f(1-f)\left[se\left(ln\widehat{OR}\right)\right]^2$, where $se\left(ln\widehat{OR}\right)$ is the reported standard error for $ln\widehat{OR}$, and f can be obtained from 1000 Genome data. The liability level effect size[6] can be approximated as $\hat{\beta}_{liability} \approx \Phi^{-1}\left[F\left(ln\left(\frac{P}{1-P}\right) + \left(ln\widehat{OR}\right)_{std}\right)\right] - \Phi^{-1}\left[F\left(ln\left(\frac{P}{1-P}\right)\right)\right]$ and the variance[7] is $var\left(\hat{\beta}_{liability}\right) = \frac{P^2(1-P)^2}{\phi^2(t)} var\left[\left(ln\widehat{OR}\right)_{std}\right]$, where Φ and ϕ are the cumulative distribution function (c.d.f) and probability density function (p.d.f) of the standard normal distribution respectively, P is the disease prevalence, F is the logistic function and $t = \Phi^{-1}(1-P)$.

Total heritability and genetic correlation

In our reciprocal joint model, $\boldsymbol{Y} = \sum_{k=1}^{K} \boldsymbol{\beta}_k^{(h)} X_k + \boldsymbol{\varepsilon}$, where X_k is the standardized genotype for the k-th SNP and $\boldsymbol{\beta}_k^{(h)}$ is a 2×1 vector of the component-dependent joint effect sizes of the k-th SNP contributing to phenotypes Y_1 and Y_2. Accordingly, the total heritability for phenotype Y_1 is calculated as follows:

$$\begin{aligned} h^2_{total(Y1)} &= var\left(\sum_{j=1}^{M^{(G_1)}} X_j^{(G_1)}\beta_{1j}^{(G_1)}\right) + var\left(\sum_{j=1}^{M^{(G_2)}} X_j^{(G_2)}\beta_{1j}^{(G_2)}\right) + var\left(\sum_{j=1}^{M^{(G_C)}} X_j^{(G_C)}\beta_{1j}^{(G_C)}\right) + var\left(\sum_{j=1}^{M^{(G_0)}} X_j^{(G_0)}\beta_{1j}^{(G_0)}\right) \\ &= \pi_1 K var\left(\beta_{1j}^{(G_1)}\right) + \pi_2 K var\left(\beta_{1j}^{(G_2)}\right) + \pi_C K var\left(\beta_{1j}^{(G_C)}\right) \\ &= \pi_1 K \frac{\sigma_1^2}{(1-\delta_{12}\delta_{21})^2} + \pi_2 K \frac{\delta_{12}^2\sigma_2^2}{(1-\delta_{12}\delta_{21})^2} + \pi_C K \frac{\sigma_{C1}^2 + \delta_{12}^2\sigma_{C2}^2}{(1-\delta_{12}\delta_{21})^2} \end{aligned}$$

where $M^{(h)}$ denotes the number of SNPs in component h, $X_j^{(h)}$ represents the standardized genotype of the j-th SNP in component h, $\beta_{1j}^{(h)}$ is the joint effect size of this j-th SNP contributing to phenotype Y_1, K is the total number of available SNPs, and $h \in (G_0, G_1, G_2, G_C)$.

Similarly, the total heritability for phenotype Y_2 is:

$$h^2_{total(Y2)} = \pi_1 K \frac{\delta_{21}^2\sigma_1^2}{(1-\delta_{12}\delta_{21})^2} + \pi_2 K \frac{\sigma_2^2}{(1-\delta_{12}\delta_{21})^2} + \pi_C K \frac{\delta_{21}^2\sigma_{C1}^2 + \sigma_{C2}^2}{(1-\delta_{12}\delta_{21})^2}$$

We derived the genetic covariance as follows:

$$cov\left(\sum_{j=1}^{M^{(G_1)}} X_j^{(G_1)}\beta_{1j}^{(G_1)} + \sum_{j=1}^{M^{(G_2)}} X_j^{(G_2)}\beta_{1j}^{(G_2)} + \sum_{j=1}^{M^{(G_C)}} X_j^{(G_C)}\beta_{1j}^{(G_C)}, \sum_{j=1}^{M^{(G_1)}} X_j^{(G_1)}\beta_{2j}^{(G_1)} + \sum_{j=1}^{M^{(G_2)}} X_j^{(G_2)}\beta_{2j}^{(G_2)} + \sum_{j=1}^{M^{(G_C)}} X_j^{(G_C)}\beta_{2j}^{(G_C)}\right)$$

$$= \pi_1 K\, cov\left(\beta_{1j}^{(G_1)}, \beta_{2j}^{(G_1)}\right) + \pi_2 K\, cov\left(\beta_{1j}^{(G_2)}, \beta_{2j}^{(G_2)}\right) + \pi_C K\, cov\left(\beta_{1j}^{(G_C)}, \beta_{2j}^{(G_C)}\right)$$

$$= \frac{\pi_1 K}{(1-\delta_{12}\delta_{21})^2}\delta_{21}\sigma_1^2 + \frac{\pi_2 K}{(1-\delta_{12}\delta_{21})^2}\delta_{12}\sigma_2^2 + \frac{\pi_C K}{(1-\delta_{12}\delta_{21})^2}\left[\delta_{21}\sigma_{C1}^2 + \delta_{12}\sigma_{C2}^2 + (1+\delta_{12}\delta_{21})\rho_{C1,C2}\right]$$

Genetic correlation (r_g) is defined as genetic covariance normalized by SNP heritabilities. Thus, the genetic correlation is written as:

$$r_g = G(\mathbf{\Theta}) = \frac{\pi_1\delta_{21}\sigma_1^2 + \pi_2\delta_{12}\sigma_2^2 + \pi_C\left[\delta_{21}\sigma_{C1}^2 + \delta_{12}\sigma_{C2}^2 + (1+\delta_{12}\delta_{21})\rho_{C1,C2}\right]}{\sqrt{(\pi_1\sigma_1^2 + \pi_2\delta_{12}^2\sigma_2^2 + \pi_C[\sigma_{C1}^2 + \delta_{12}^2\sigma_{C2}^2)]) \cdot (\pi_1\delta_{21}^2\sigma_1^2 + \pi_2\sigma_2^2 + \pi_C[\delta_{21}^2\sigma_{C1}^2 + \sigma_{C2}^2])}}$$

Here, $\mathbf{\Theta}$ is the vector of random variables in function $G(\mathbf{\Theta})$. The variance of r_g could be approximated by the Delta method as $var(r_g) \approx \nabla G(\mathbf{\Theta})^T cov(\mathbf{\Theta}) \nabla G(\mathbf{\Theta})$, where $\nabla G(\mathbf{\Theta})$ is the gradient of $G(\mathbf{\Theta})$ at the estimated values and $cov(\mathbf{\Theta})$ is the variance-covariance matrix of $\mathbf{\Theta}$.

Supplementary References

1. Yang, J. *et al.* Conditional and joint multiple-SNP analysis of GWAS summary statistics identifies additional variants influencing complex traits. *Nat Genet* **44**, 369-75, S1-3 (2012).
2. Zhang, Y., Qi, G., Park, J.H. & Chatterjee, N. Estimation of complex effect-size distributions using summary-level statistics from genome-wide association studies across 32 complex traits. *Nat Genet* (2018).
3. Varin, C., Reid, N. & Firth, D. An overview of composite likelihood methods. *Statistica Sinica* **21**, 5-42 (2011).
4. Batram, M. & Bauer, D. Model selection and model averaging in MACML-estimated MNP models. arXiv:1704.00183 (2017).
5. Buckland, S.T., Burnham, K.P. & Augustin, N.H. Model Selection: An Integral Part of Inference. *Biometrics* **53**, 603-618 (1997).
6. Gillett, A.C., Vassos, E. & Lewis, C.M. Transforming Summary Statistics from Logistic Regression to the Liability Scale: Application to Genetic and Environmental Risk Scores. *Human Heredity* **83**, 210-224 (2018).
7. Wu, T. & Sham, P.C. On the Transformation of Genetic Effect Size from Logit to Liability Scale. *Behav Genet* **51**, 215-222 (2021).

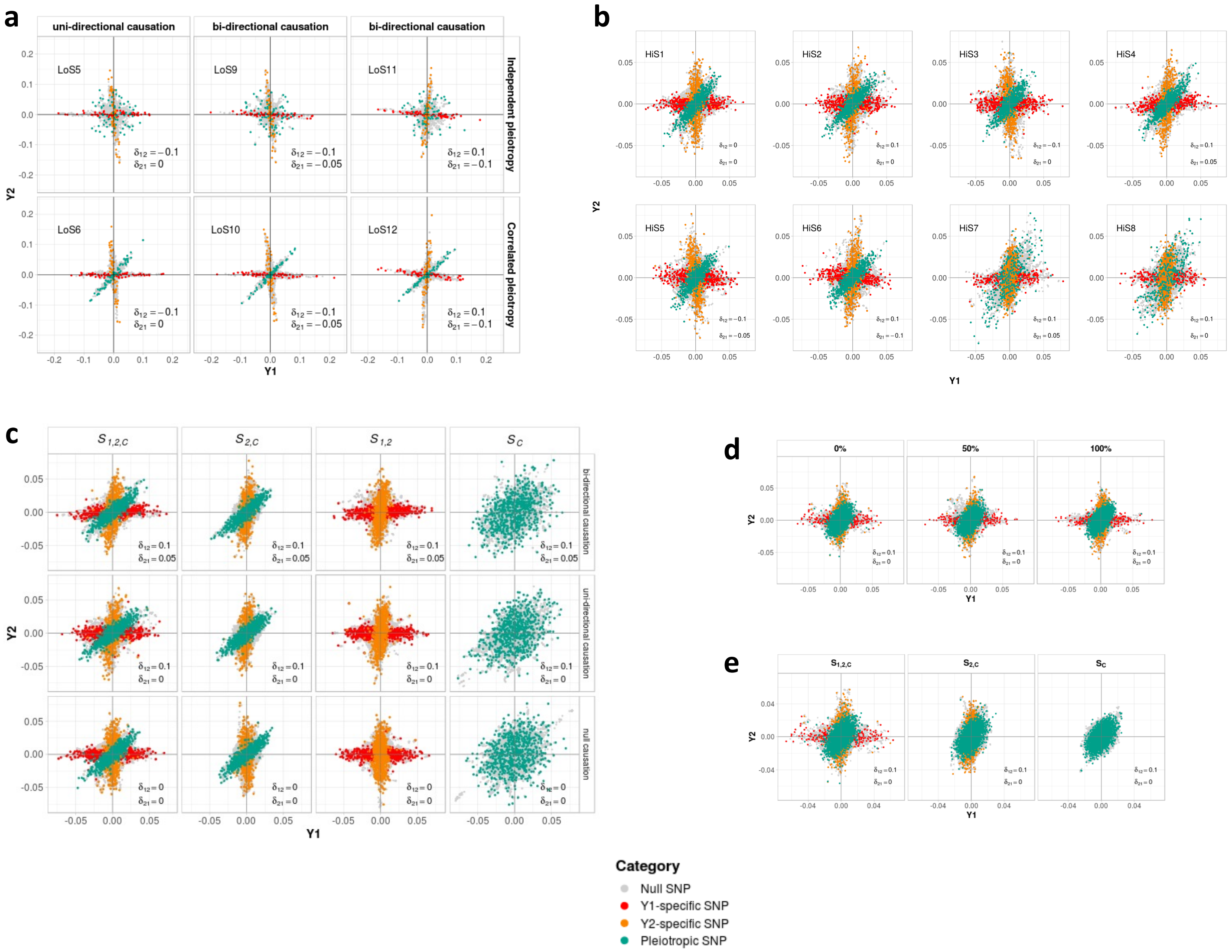

Supplementary Figure 1. Illustration of simulation scenarios. x- and y-axis are the standardized effect size estimates for GWAS Y_1 and Y_2 , respectively. Green, red, orange and grey points represent pleiotropic, Y_1-specific, Y_2-specific and null SNPs in the simulation. The reciprocal causal effects (δ_{12} and δ_{21}) are shown in each plot. **a,** The mixing proportion for each component is set as: $\pi_1 = \pi_2 = \pi_c = 1\times10^{-4}$. For independent pleiotropy, $\rho_{C1,C2} = 0.0$; for correlated pleiotropy, $\rho_{C1,C2} = 0.1$. **b,** Scatterplots of representative simulated high polygenicity scenarios ($\pi_1 = \pi_2 = \pi_c = 1\times10^{-3}$). **c**, Scatterplots of representative simulated high polygenicity sub-model scenarios ($\pi_1 = \pi_2 = \pi_c = 1\times10^{-3}$). $s_{1,2,C}$ is the full model scenario where all three non-null components are present; $s_{2,C}$ is the sub-model scenario where Y_1-specific component is absent; $s_{1,2}$ is the sub-model scenario where pleiotropic component is absent; s_C is the sub-model scenario where both Y_1- and Y_2-specific components are absent. For null causation, $\delta_{12} = \delta_{21} = 0.0$; for uni-directional causation, $\delta_{12} = 0.1$ and $\delta_{21} = 0.0$; for bi-directional causation, $\delta_{12} = 0.1$ and $\delta_{21} = 0.05$. **d**, Scatterplots of representative unbalanced pleiotropy scenarios under different levels of sample overlapping (from 0% to 100%). In these simulations, $\delta_{12} = 0.1$ and $\delta_{21} = 0.0$; the effects of genetic components are unbalanced. **e**, Scatterplots of representative unbalanced pleiotropy sub-model scenarios ($s_{2,C}$ and s_C). In sub-model simulations, $\delta_{12} = 0.1$ and $\delta_{21} = 0.0$; the effects of genetic components are unbalanced. Simulation settings for each scenario are shown in Supplementary Table S1.

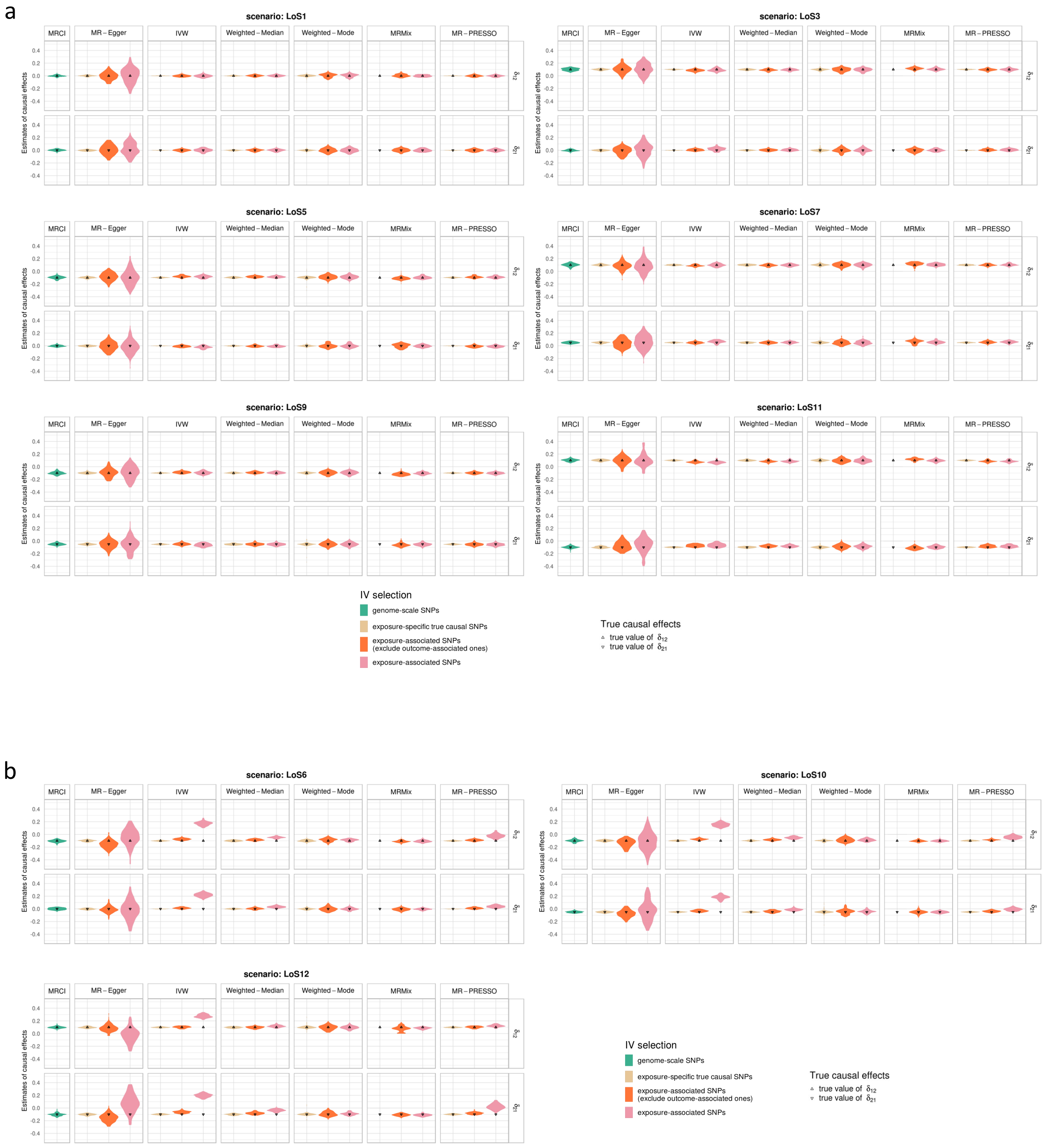

Supplementary Figure 2. Comparison of the reciprocal causal estimates by our method and instrumental variables (IV)-based MR methods. a, estimates under independent pleiotropy simulations ($\rho_{C1,C2} = 0.0$); **b** estimates under correlated pleiotropy simulations ($\rho_{C1,C2} = 0.1$). Our method takes whole-genome scale SNPs for estimation. For MR methods, IVs are selected in three ways: (1) use the exposure-specific true causal SNPs as IVs; (2) use exposure-associated SNPs (p-value < 5×10^{-8}) after clumping but exclude potential outcome-associated SNPs (p-value < 5×10^{-5} with outcome); (3) use significant exposure-associated SNPs after clumping regardless of their association with outcome. The true values of δ_{12}/δ_{21} are indicated by up-/down-pointing triangles, respectively. Simulations were performed under low polygenicity settings ($\pi_1 = \pi_2 = \pi_c = 1\times10^{-4}$).

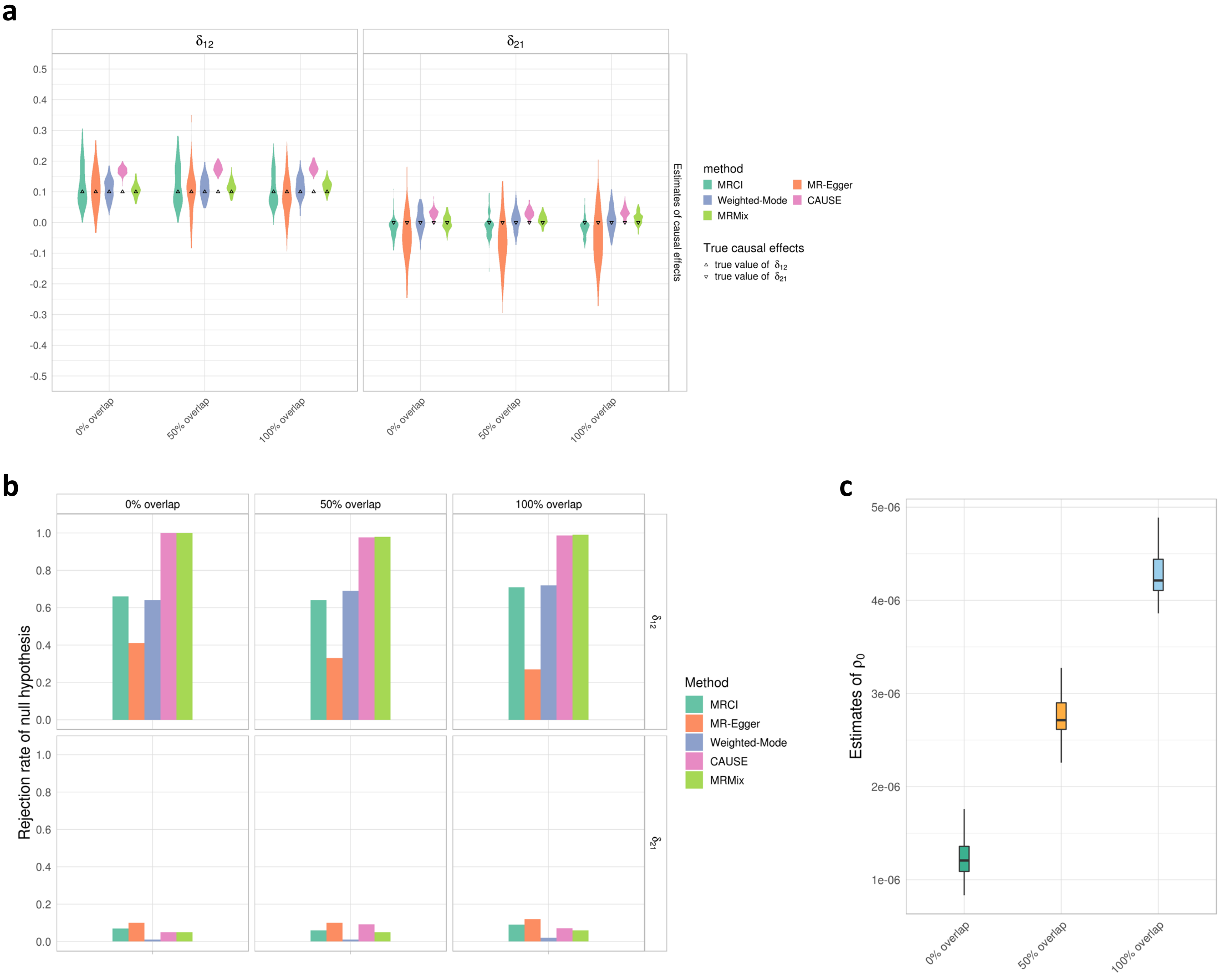

Supplementary Figure 3. Estimation comparison using different methods under various sample overlapping conditions. Data were from simulations with 0%, 50% and 100% sample overlapping, respectively. The causal effects were set as: $\delta_{12} = 0.1$ and $\delta_{21} = 0.0$. **a**, shows causal estimates from MRCI and selected standard MR methods. MRCI shows nearly unbiased estimates regardless of sample overlapping. **b**, type I error rate (δ_{21}) and power (δ_{12}) of estimation between MRCI and selected existing MR methods. MRCI shows adequate power and controlled type I error rate under different sample overlapping scenarios. **c**, nuisance parameter ρ_0 in our model could reflect the degree of sample overlapping, i.e., estimate of ρ_0 increases as the degree of sample overlapping increase. In the simulation, the mixing proportions of π_1, π_2 and π_C were set as 5×10^{-4}, 2×10^{-3} and 5×10^{-3} respectively; the heritabilities of h_1^2, h_2^2 , h_{C1}^2 , and h_{C2}^2 were set as 0.2, 0.3, 0.1 and 0.2, respectively; $\rho_{C1,C2}$ was set as 0.1.

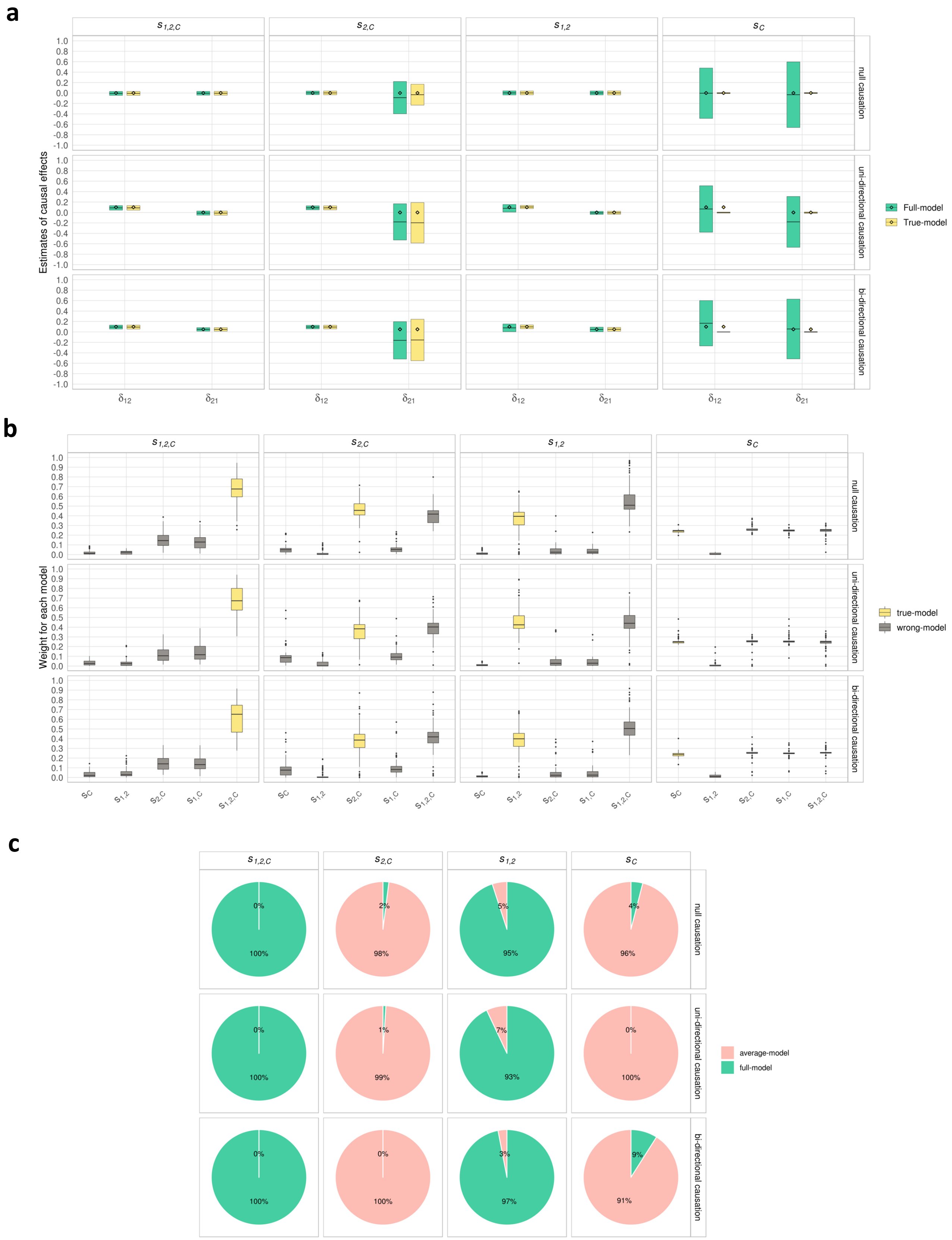

Supplementary Figure 4. Estimation using model averaging in four simulated scenarios ($s_{1,2,C}, s_{2,C}, s_{1,2}$ **and** s_C**).** In each scenario, we considered null ($\delta_{12} = \delta_{21} = 0.0$), uni-directional ($\delta_{12} = 0.1$ and $\delta_{12} = 0.0$) and bi-directional ($\delta_{12} = 0.1$ and $\delta_{12} = 0.05$) causations. **a**, estimate comparison between the full model and the true model. The full model could not always give accurate estimates in sub-model scenarios (e.g., $s_{2,C}$ and s_C). The estimates in the plots are shown as $mean \pm 2SD$. The black diamonds show the true values of δ_{12} and δ_{21}. **b**, weight for each model during model averaging. This averaging strategy largely gives higher weights to the true-model. **c**, frequency of full- or averaged-model being selected as the final estimates. Results suggest that averaged model is more favorable when the exposure-specific component is absent (e.g., $s_{2,C}$ and s_C). In the simulations, the mixing proportions of the present component were set as 1×10^{-3}; the heritabilities contributed by Y_1-specific, Y_2-specific and pleiotropic SNPs (if present in the sub-model scenario) were set as 0.3, 0.3 and 0.1, respectively; $\rho_{C1,C2}$ was set as 0.1.

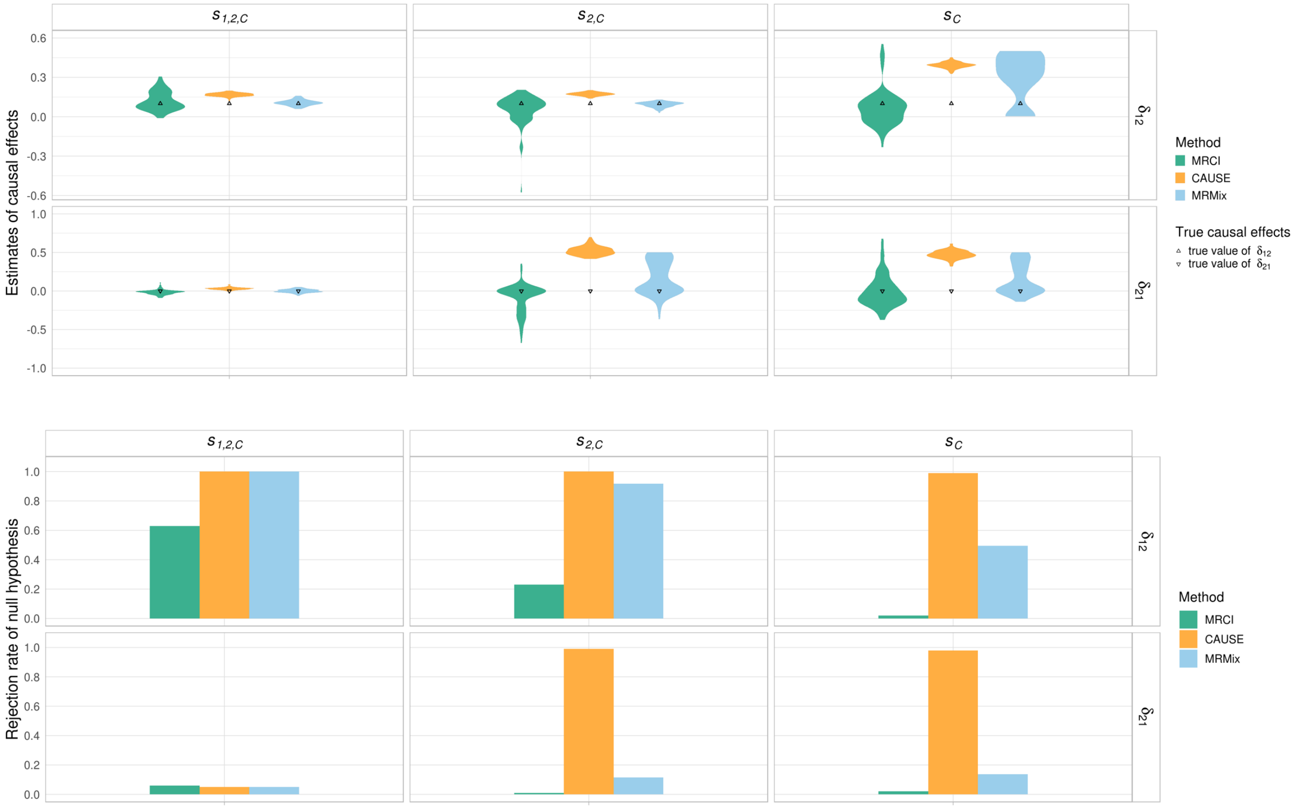

Supplementary Figure 5. Estimation of unbalanced pleiotropy simulation under three sub-model scenarios ($s_{1,2,C}$, $s_{2,C}$ and s_C). The final estimates of MRCI under $S_{1,2,C}$, $S_{2,C}$ and S_C scenarios were still around the true values and the type I error rate was well-controlled. When one or two components were missing ($S_{2,C}$ and S_C), CAUSE and MRMix generated biased estimates. In each scenario, the true values of δ_{12} and δ_{21} were set as 0.1 and 0.0 (represented by triangles), respectively; the mixing proportions of π_1, π_2 and π_C were set as 5×10^{-4}, 2×10^{-3} and 5×10^{-3} respectively; the heritabilities of h_1^2, h_2^2 , h_{C1}^2 , and h_{C2}^2 were set as 0.2, 0.3, 0.1 and 0.2, respectively; $\rho_{C1,C2}$ was set as 0.1. The corresponding parameters of a component were set as 0 if the component was absent in the sub-model scenario.

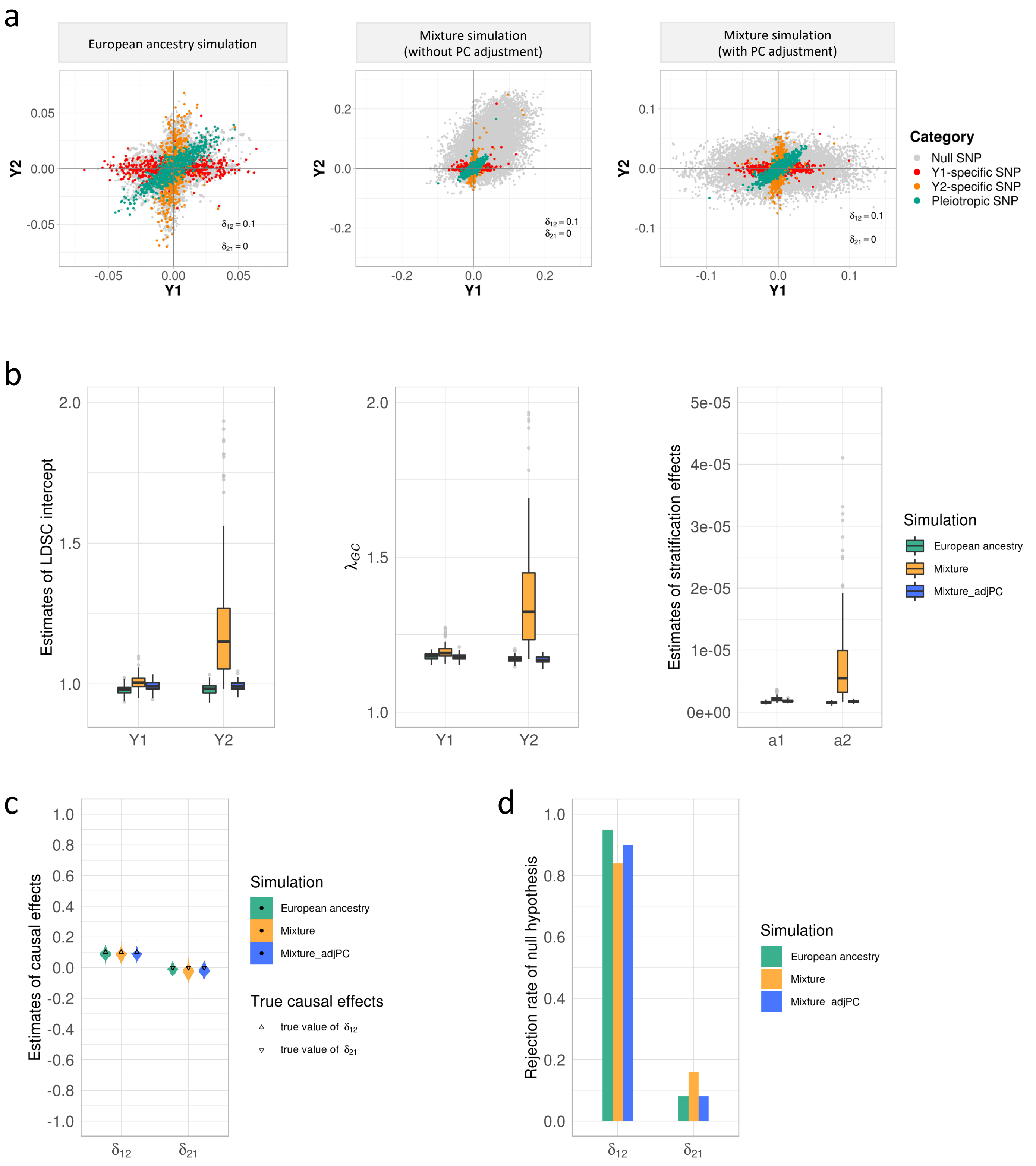

Supplementary Figure 6. Simulations under stratified population. The "European ancestry" simulations only included European ancestry individuals while the 'mixture' simulations included 1% and 5% non-European ancestry individuals for Y_1 and Y_2, respectively. **a**, Representative scatterplots of the European ancestry and the mixture simulations. For the mixture simulations, we compared the summary statistics with or without adjusting for the top 10 principal components (PCs). **b**, estimates of LDSC intercept, genomic control (λ_{GC}) and estimates of stratification factors in our model were shown in the plots. The stratification factor parameters in our model reflected the increased level of population stratification and behaved similarly to the other two indices. **c**, Causal estimates of our method in stratified simulations were still near the true values. **d**, Type I error rate (for δ_{21}) was still well-controlled if the stratification effects could be adjusted. In the simulations, $\delta_{12} = 0.1$ and $\delta_{21} = 0.0$; $\pi_1 = \pi_2 = \pi_C = 1{\times}10^{-3}$; $h_1^2 = h_2^2 = 0.3$, $h_{C1}^2 = h_{C2}^2 = 0.1$ and $\rho_{C1,C2} = 0.1$.

Supplementary Table 1. Parameter settings for various simulation scenarios. In the table, the column 'simID' shows the names of the corresponding simulated scenarios. $s_{1,2,C}$ is the full model scenario where all three non-null components (i.e. Y_1-specific, Y_2-specific and pleiotropic components) are present, $s_{2,C}$ is the sub-model scenario where Y_1-specific component is absent, $s_{1,2}$ is the sub-model scenario where pleiotropic component is absent and s_C is the sub-model scenario where both Y_1- and Y_2-specific components are absent. π_1, π_2 and π_C denote the mixing proportions of Y_1-specific, Y_2-specific and pleiotropic component, respectively. h_1^2, h_2^2, h_{C1}^2, h_{C2}^2 denote the direct heritabilities contributed by Y_1-specific, Y_2-specific and pleiotropic components to the Y_1, Y_2 phenotypes. $\rho_{C1,C2}$ denotes the covariance between pleiotropic effects. δ_{12} and δ_{21} denote the causal effects of Y_2->Y_1 and Y_1->Y_2. For null causation, $\delta_{12} = \delta_{21} = 0.0$; for uni-directional causation, one of the causal effects is zero and the other one is non-zero; for bi-directional causation, both δ_{12} and δ_{21} are non-zero. The sample size and percentage of sample overlapping in simulation are also listed.

group	causation	simID	π_1	π_2	π_C	h_1^2	h_2^2	h_{C1}^2	h_{C2}^2	$\rho_{C1,C2}$	δ_{12}	δ_{21}	sample size (Y_1/ Y_2)	sample overlapping
Low Polygenicity ($s_{1,2,C}$ model)	null	LoS1	1e-4	1e-4	1e-4	0.3	0.3	0.1	0.1	0.0	0.0	0.0	50K / 50K	100%
		LoS2	1e-4	1e-4	1e-4	0.3	0.3	0.1	0.1	0.1	0.0	0.0	50K / 50K	100%
	uni-directional	LoS3	1e-4	1e-4	1e-4	0.3	0.3	0.1	0.1	0.0	0.1	0.0	50K / 50K	100%
		LoS4	1e-4	1e-4	1e-4	0.3	0.3	0.1	0.1	0.1	0.1	0.0	50K / 50K	100%
		LoS5	1e-4	1e-4	1e-4	0.3	0.3	0.1	0.1	0.0	-0.1	0.0	50K / 50K	100%
		LoS6	1e-4	1e-4	1e-4	0.3	0.3	0.1	0.1	0.1	-0.1	0.0	50K / 50K	100%
	bi-directional	LoS7	1e-4	1e-4	1e-4	0.3	0.3	0.1	0.1	0.0	0.1	0.05	50K / 50K	100%
		LoS8	1e-4	1e-4	1e-4	0.3	0.3	0.1	0.1	0.1	0.1	0.05	50K / 50K	100%
		LoS9	1e-4	1e-4	1e-4	0.3	0.3	0.1	0.1	0.0	-0.1	-0.05	50K / 50K	100%
		LoS10	1e-4	1e-4	1e-4	0.3	0.3	0.1	0.1	0.1	-0.1	-0.05	50K / 50K	100%
		LoS11	1e-4	1e-4	1e-4	0.3	0.3	0.1	0.1	0.0	0.1	-0.1	50K / 50K	100%
		LoS12	1e-4	1e-4	1e-4	0.3	0.3	0.1	0.1	0.1	0.1	-0.1	50K / 50K	100%
High Polygenicity ($s_{1,2,C}$ model)	null	HiS1	1e-3	1e-3	1e-3	0.3	0.3	0.1	0.1	0.1	0.0	0.0	50K / 50K	100%
	uni-directional	HiS2	1e-3	1e-3	1e-3	0.3	0.3	0.1	0.1	0.1	0.1	0.0	50K / 50K	100%
		HiS3	1e-3	1e-3	1e-3	0.3	0.3	0.1	0.1	0.1	-0.1	0.0	50K / 50K	100%
	bi-directional	HiS4	1e-3	1e-3	1e-3	0.3	0.3	0.1	0.1	0.1	0.1	0.05	50K / 50K	100%
		HiS5	1e-3	1e-3	1e-3	0.3	0.3	0.1	0.1	0.1	-0.1	-0.05	50K / 50K	100%
		HiS6	1e-3	1e-3	1e-3	0.3	0.3	0.1	0.1	0.1	0.1	-0.1	50K / 50K	100%
		HiS7	5e-4	2e-3	5e-3	0.2	0.3	0.1	0.2	0.1	0.1	0.05	50K / 50K	100%
	uni-directional	HiS8*	5e-4	2e-3	5e-3	0.2	0.3	0.1	0.2	0.1	0.1	0.0	50K / 45K	0%
High Polygenicity (sub-models)	null	$s_{2,c}$	0.0	1e-3	1e-3	0.0	0.3	0.1	0.1	0.1	0.0	0.0	50K / 50K	100%
		$s_{1,2}$	1e-3	1e-3	0.0	0.3	0.3	0.0	0.0	0.0	0.0	0.0	50K / 50K	100%
		s_c	0.0	0.0	1e-3	0.0	0.0	0.3	0.4	0.1	0.0	0.0	50K / 50K	100%
	uni-directional	$s_{2,c}$	0.0	1e-3	1e-3	0.0	0.3	0.1	0.1	0.1	0.1	0.0	50K / 50K	100%
		$s_{1,2}$	1e-3	1e-3	0.0	0.3	0.3	0.0	0.0	0.0	0.1	0.0	50K / 50K	100%
		s_c	0.0	0.0	1e-3	0.0	0.0	0.3	0.4	0.1	0.1	0.0	50K / 50K	100%
	bi-directional	$s_{2,c}$	0.0	1e-3	1e-3	0.0	0.3	0.1	0.1	0.1	0.1	0.05	50K / 50K	100%
		$s_{1,2}$	1e-3	1e-3	0.0	0.3	0.3	0.0	0.0	0.0	0.1	0.05	50K / 50K	100%
		s_c	0.0	0.0	1e-3	0.0	0.0	0.3	0.4	0.1	0.1	0.05	50K / 50K	100%
High Polygenicity (small sample sizes)	bi-directional	SS1	1e-3	1e-3	1e-3	0.3	0.3	0.1	0.1	0.1	0.1	0.05	20K / 20K	100%
		SS2#	1e-3	1e-3	1e-3	0.3	0.3	0.1	0.1	0.1	0.1	0.05	50K / 20K	100%
	uni-directional	SS3	1e-3	1e-3	1e-3	0.3	0.3	0.1	0.1	0.1	0.1	0.0	20K / 20K	100%
	null	SS4	1e-3	1e-3	1e-3	0.3	0.3	0.1	0.1	0.1	0.0	0.0	20K / 20K	100%
Unbalanced Genetic Components ($s_{1,2,C}$ model)	uni-directional	Unbalance1	5e-4	2e-3	5e-3	0.2	0.3	0.1	0.2	0.1	0.1	0.0	50K / 50K	0%
		Unbalance2	5e-4	2e-3	5e-3	0.2	0.3	0.1	0.2	0.1	0.1	0.0	50K / 50K	50%
		Unbalance3	5e-4	2e-3	5e-3	0.2	0.3	0.1	0.2	0.1	0.1	0.0	50K / 50K	100%
Unbalanced Genetic Components (sub-model)	uni-directional	$s_{1,2,c}$	5e-4	2e-3	5e-3	0.2	0.3	0.1	0.2	0.1	0.1	0.0	50K / 50K	0%
		$s_{2,c}$	0.0	2e-3	5e-3	0.0	0.3	0.1	0.2	0.1	0.1	0.0	50K / 50K	0%
		s_c	0.0	0	5e-3	0.0	0.3	0.1	0.2	0.1	0.1	0.0	50K / 50K	0%
Stratified population	uni-directional	Strat$	1e-3	1e-3	1e-3	0.3	0.3	0.1	0.1	0.1	0.1	0.0	50K / 50K	95%

* In this scenario, Y_1 is a continuous trait and Y_2 is a binary trait. The prevalence and case:control ratio for Y_2 were set as 5% and 1:2, respectively. h^2 values for Y_2 were defined on a liability scale.

\# The 20K individuals were completely included in the 50K individuals

$ The proportions of non-European ancestry individuals in simulation were 1% and 5% for Y_1 and Y_2 respectively.

Supplementary Table 2. Comparison of Type I error rate from different methods under the null hypothesis ($\boldsymbol{\delta_{12} = \delta_{21} = 0.0}$) from 100 simulations. When using exposure-specific true causal SNPs as instrumental variables (IVs), both our method and other MR methods show well-controlled Type I error rate. When using significant IVs from GWAS summary data, for simulations with independent pleiotropy, our method and most MR methods could produce a reasonable Type I error rate at the nominal level of $\alpha = 0.05$. However, for simulations with correlated pleiotropy, many of the selected MR methods show an inflated Type I error rate especially when exclusion restriction in outcome data is not properly performed, while our method can maintain good control of Type I error rate. In the table, the χ^2 is calculated as $(\frac{estimate}{standard\ error})^2$. For independent pleiotropy $\rho_{C1,C2} = 0.0$; for correlated pleiotropy $\rho_{C1,C2} = 0.1$. See Supplementary Table S1 for detailed settings of each simulated scenario.

IVs selection	Method	LoS1 (independent pleiotropy)				LoS2 (correlated pleiotropy)			
		$\boldsymbol{\delta_{12} = 0.0}$		$\boldsymbol{\delta_{21} = 0.0}$		$\boldsymbol{\delta_{12} = 0.0}$		$\boldsymbol{\delta_{21} = 0.0}$	
		Mean χ^2 (SD)	Type I error rate	Mean χ^2 (SD)	Type I error rate	Mean χ^2 (SD)	Type I error rate	Mean χ^2 (SD)	Type I error rate
**N/A (Genome-scale SNPs)**	MRCI	0.93 (1.36)	0.05	0.95 (1.31)	0.05	1.24 (1.85)	0.08	0.93 (1.45)	0.04
**exposure-specific true causal SNPs**	MR-Egger	0.57 (0.80)	0.00	0.61 (0.81)	0.01	0.53 (0.85)	0.01	0.55 (0.72)	0.00
	Weighted Median	0.35 (0.53)	0.00	0.41 (0.60)	0.01	0.45 (0.61)	0.00	0.48 (0.74)	0.01
	IVW	0.10 (0.09)	0.00	0.10 (0.10)	0.00	0.13 (0.11)	0.00	0.11 (0.10)	0.00
	Weighted Mode	0.19 (0.31)	0.00	0.15 (0.26)	0.00	0.24 (0.43)	0.00	0.24 (0.50)	0.00
	MRMix*	N/A	N/A	N/A	N/A	N/A	N/A	N/A	N/A
	MR-PRESSO	0.10 (0.10)	0.00	0.11 (0.11)	0.00	0.14 (0.13)	0.00	0.11 (0.11)	0.00
**significant exposure-associated SNPs excluding potential outcome-associated SNPs**	MR-Egger	0.91 (1.18)	0.02	1.27 (1.49)	0.07	1.25 (1.94)	0.07	1.43 (2.20)	0.09
	Weighted Median	0.76 (1.03)	0.02	1.04 (1.55)	0.06	0.90 (1.25)	0.04	0.81 (1.13)	0.03
	IVW	1.15 (2.14)	0.05	1.33 (1.98)	0.09	1.98 (2.40)	0.15	2.06 (2.47)	0.20
	Weighted Mode	0.40 (0.43)	0.00	0.51 (0.66)	0.01	0.54 (0.79)	0.01	0.46 (0.61)	0.00
	MRMix	0.57 (0.88)	0.01	0.72 (1.14)	0.04	0.63 (1.74)	0.01	0.48 (0.92)	0.02
	MR-PRESSO	1.37 (2.31)	0.09	1.91 (2.39)	0.15	1.70 (2.22)	0.12	1.71 (2.27)	0.14
**significant exposure-associated SNPs with no exclusion in outcome**	MR-Egger	1.30 (1.66)	0.07	0.95 (1.34)	0.03	0.88 (1.31)	0.03	1.19 (1.30)	0.07
	Weighted Median	0.75 (1.04)	0.02	1.10 (1.57)	0.05	5.36 (3.79)	0.57	4.82 (4.18)	0.50
	IVW	0.82 (1.31)	0.03	1.14 (1.56)	0.09	55.06 (10.38)	1.00	56.49 (11.87)	1.00
	Weighted Mode	0.40 (0.45)	0.00	0.51 (0.59)	0.00	0.56 (0.78)	0.02	0.62 (0.78)	0.01
	MRMix	0.68 (0.78)	0.00	1.06 (1.25)	0.06	0.69 (0.99)	0.01	0.70 (1.16)	0.02
	MR-PRESSO	1.35 (2.55)	0.08	1.76 (2.26)	0.14	11.95 (7.33)	0.90	13.50 (8.84)	0.92

(Note: *Not applied to MRMix due to its mixture-model assumption.)

Supplementary Table 3. Results of hypothesis testing under uni-directional causations ($\boldsymbol{\delta_{12} = 0.1}$, $\boldsymbol{\delta_{21} = 0.0}$) from 100 simulations. In the table, the χ^2 is calculated as $(\frac{estimate}{standard\ error})^2$. For independent pleiotropy $\rho_{C1,C2} = 0.0$; for correlated pleiotropy $\rho_{C1,C2} = 0.1$. See Supplementary Table S1 for detailed settings of each simulated scenario.

IVs selection	Method	LoS3 (independent pleiotropy)				LoS4 (correlated pleiotropy)			
		$\delta_{12} = 0.1$		$\delta_{21} = 0.0$		$\delta_{12} = 0.1$		$\delta_{21} = 0.0$	
		Mean χ^2 (SD)	Power	Mean χ^2 (SD)	Type I error rate	Mean χ^2 (SD)	Power	Mean χ^2 (SD)	Type I error rate
N/A	MRCI	47.08 (50.11)	0.97	1.01 (1.39)	0.03	68.27 (55.30)	0.93	0.92 (1.07)	0.02
**exposure-specific true causal SNPs**	MR-Egger	55.84 (16.35)	1.00	0.60 (0.77)	0.01	51.47 (16.65)	1.00	0.53 (0.64)	0.00
	Weighted Mode	27.45 (25.05)	0.67	0.22 (0.43)	0.00	24.53 (24.35)	0.66	0.20 (0.35)	0.00
	MRMix*	N/A	N/A	N/A	N/A	N/A	N/A	N/A	N/A
**significant exposure-associated SNPs excluding potential outcome-associated SNPs**	MR-Egger	4.05 (3.93)	0.39	0.84 (1.10)	0.04	2.90 (2.89)	0.24	1.14 (1.37)	0.08
	Weighted Mode	4.82 (2.86)	0.58	0.53 (0.72)	0.00	4.43 (2.94)	0.48	0.40 (0.55)	0.00
	MRMix	25.85 (19.26)	0.89	0.83 (1.25)	0.05	12.49 (33.18)	0.49	0.64 (1.00)	0.01
**significant exposure-associated SNPs with no exclusion in outcome**	MR-Egger	1.97 (2.13)	0.20	1.02 (1.30)	0.05	0.99 (1.27)	0.04	1.90 (2.12)	0.15
	Weighted Mode	5.92 (3.39)	0.66	0.58 (0.81)	0.01	10.57 (5.53)	0.88	0.53 (0.72)	0.00
	MRMix	25.39 (12.25)	0.95	0.87 (1.10)	0.02	23.06 (18.32)	0.71	0.95 (1.38)	0.06

(Note: *Not applied to MRMix due to its mixture-model assumption.)

Supplementary Table 4. Results of hypothesis testing under uni-directional causations ($\boldsymbol{\delta_{12} = -0.1,\ \delta_{21} = 0.0}$) from 100 simulations. In the table, the χ^2 is calculated as $(\frac{estimate}{standard\ error})^2$. For independent pleiotropy $\rho_{C1,C2} = 0.0$; for correlated pleiotropy $\rho_{C1,C2} = 0.1$. See Supplementary Table S1 for detailed settings of each simulated scenario.

IVs selection	Method	LoS5 (independent pleiotropy)				LoS6 (correlated pleiotropy)			
		$\boldsymbol{\delta_{12} = -0.1}$		$\boldsymbol{\delta_{21} = 0.0}$		$\boldsymbol{\delta_{12} = -0.1}$		$\boldsymbol{\delta_{21} = 0.0}$	
		Mean χ^2 (SD)	Power	Mean χ^2 (SD)	Type I error rate	Mean χ^2 (SD)	Power	Mean χ^2 (SD)	Type I error rate
N/A	MRCI	42.88 (43.48)	0.92	1.00 (1.19)	0.02	60.47 (38.86)	0.97	1.18 (1.29)	0.06
**exposure-specific true causal SNPs**	MR-Egger	51.19 (15.55)	1.00	0.65 (0.88)	0.01	56.23 (18.47)	1.00	0.61 (0.76)	0.01
	Weighted Mode	24.93 (24.18)	0.69	0.22 (0.42)	0.00	30.21 (29.25)	0.71	0.27 (0.49)	0.00
	MRMix*	N/A	N/A	N/A	N/A	N/A	N/A	N/A	N/A
**significant exposure-associated SNPs excluding potential outcome-associated SNPs**	MR-Egger	2.85 (3.21)	0.25	1.13 (1.39)	0.07	6.74 (5.00)	0.68	0.81 (1.37)	0.06
	Weighted Mode	4.08 (2.68)	0.49	0.42 (0.56)	0.00	3.95 (2.55)	0.39	0.41 (0.53)	0.00
	MRMix	21.71 (16.67)	0.92	1.07 (1.53)	0.07	30.68 (16.39)	0.92	0.96 (3.38)	0.03
**significant exposure-associated SNPs with no exclusion in outcome**	MR-Egger	2.23 (2.58)	0.21	0.94 (1.40)	0.03	0.96 (1.74)	0.04	1.03 (1.42)	0.07
	Weighted Mode	5.08 (3.23)	0.57	0.42 (0.52)	0.00	12.26 (5.83)	1.00	0.46 (0.73)	0.01
	MRMix	22.44 (10.94)	0.99	0.88 (1.08)	0.03	34.04 (24.78)	0.72	0.51 (0.80)	0.00

(Note: *Not applied to MRMix due to its mixture-model assumption.)

Supplementary Table 5. Results of hypothesis testing under bi-directional causations ($\boldsymbol{\delta_{12} = 0.1}$, $\boldsymbol{\delta_{21} = 0.05}$) from 100 simulations. In the table, the χ^2 is calculated as $(\frac{estimate}{standard\ error})^2$. For independent pleiotropy $\rho_{C1,C2} = 0.0$; for correlated pleiotropy $\rho_{C1,C2} = 0.1$. See Supplementary Table S1 for detailed settings of each simulated scenario.

**IVs selection**	**Method**	**LoS7 (independent pleiotropy)**				**LoS8 (correlated pleiotropy)**			
		$\boldsymbol{\delta_{12} = 0.1}$		$\boldsymbol{\delta_{21} = 0.05}$		$\boldsymbol{\delta_{12} = 0.1}$		$\boldsymbol{\delta_{21} = 0.05}$	
		Mean χ^2 (SD)	Power	Mean χ^2 (SD)	Power	Mean χ^2 (SD)	Power	Mean χ^2 (SD)	Power
N/A	MRCI	53.53 (57.73)	0.89	15.95 (13.38)	0.85	78.61 (65.79)	1.00	18.94 (18.93)	0.86
**exposure-specific true causal SNPs**	MR-Egger	55.43 (18.26)	1.00	14.33 (7.30)	0.94	52.65 (14.71)	1.00	14.15 (7.90)	0.94
	Weighted Mode	26.46 (24.41)	0.65	6.35 (7.67)	0.48	22.83 (25.18)	0.61	6.45 (7.19)	0.50
	MRMix*	N/A	N/A	N/A	N/A	N/A	N/A	N/A	N/A
**significant exposure-associated SNPs excluding potential outcome-associated SNPs**	MR-Egger	2.87 (2.89)	0.27	1.42 (1.88)	0.08	2.82 (2.87)	0.31	1.19 (1.55)	0.05
	Weighted Mode	4.85 (2.71)	0.62	1.50 (1.63)	0.08	4.77 (3.00)	0.53	1.95 (1.70)	0.14
	MRMix	26.54 (23.35)	0.89	6.50 (5.82)	0.62	9.23 (12.29)	0.59	3.58 (8.81)	0.23
**significant exposure-associated SNPs with no exclusion in outcome**	MR-Egger	1.82 (2.67)	0.13	1.45 (1.90)	0.12	1.45 (1.75)	0.08	2.13 (2.18)	0.19
	Weighted Mode	6.12 (3.35)	0.74	1.86 (1.99)	0.16	12.89 (5.44)	0.97	4.91 (3.38)	0.60
	MRMix	27.25 (13.61)	1.00	8.25 (6.50)	0.72	25.08 (18.02)	0.74	5.57 (5.82)	0.51

(Note: *Not applied to MRMix due to its mixture-model assumption.)

Supplementary Table 6. Results of hypothesis testing under bi-directional causations ($\boldsymbol{\delta_{12} = -0.1,\ \delta_{21} = -0.05}$) from 100 simulations. In the table, the χ^2 is calculated as $(\frac{estimate}{standard\ error})^2$. For independent pleiotropy $\rho_{C1,C2} = 0.0$; for correlated pleiotropy $\rho_{C1,C2} = 0.1$. See Supplementary Table S1 for detailed settings of each simulated scenario.

IVs selection	Method	LoS9 (independent pleiotropy)				LoS10 (correlated pleiotropy)			
		$\boldsymbol{\delta_{12} = -0.1}$		$\boldsymbol{\delta_{21} = -0.05}$		$\boldsymbol{\delta_{12} = -0.1}$		$\boldsymbol{\delta_{21} = -0.05}$	
		Mean χ^2 (SD)	Power	Mean χ^2 (SD)	Power	Mean χ^2 (SD)	Power	Mean χ^2 (SD)	Power
N/A	MRCI	56.37 (53.02)	0.95	13.83 (12.20)	0.76	72.76 (59.33)	0.92	17.39 (13.82)	0.83
**exposure-specific true causal SNPs**	MR-Egger	53.13 (17.37)	1.00	14.79 (7.22)	0.96	54.78 (16.39)	1.00	13.84 (6.82)	0.95
	Weighted Mode	28.03 (24.19)	0.76	7.44 (6.73)	0.59	25.40 (25.22)	0.65	6.96 (7.97)	0.48
	MRMix*	N/A	N/A	N/A	N/A	N/A	N/A	N/A	N/A
**significant exposure-associated SNPs excluding potential outcome-associated SNPs**	MR-Egger	3.29 (3.41)	0.33	1.64 (2.21)	0.14	5.92 (5.21)	0.57	2.35 (2.50)	0.19
	Weighted Mode	4.57 (2.75)	0.54	1.59 (1.52)	0.09	4.02 (2.88)	0.46	1.43 (1.51)	0.07
	MRMix	26.96 (19.29)	0.92	5.15 (6.08)	0.45	23.93 (16.06)	0.90	6.02 (8.09)	0.57
**significant exposure-associated SNPs with no exclusion in outcome**	MR-Egger	1.84 (2.37)	0.18	1.37 (1.95)	0.13	1.56 (2.00)	0.12	1.10 (1.39)	0.05
	Weighted Mode	5.55 (3.08)	0.65	1.85 (1.60)	0.14	9.91 (4.94)	0.92	2.53 (2.52)	0.21
	MRMix	26.37 (12.06)	0.99	7.32 (6.08)	0.66	31.00 (21.36)	0.74	7.38 (6.91)	0.60

(Note: *Not applied to MRMix due to its mixture-model assumption.)

Supplementary Table 7. Results of hypothesis testing under bi-directional causations ($\boldsymbol{\delta_{12} = 0.1}$, $\boldsymbol{\delta_{21} = -0.1}$) from 100 simulations. In the table, the χ^2 is calculated as $(\frac{estimate}{standard\ error})^2$. For independent pleiotropy $\rho_{C1,C2} = 0.0$; for correlated pleiotropy $\rho_{C1,C2} = 0.1$. See Supplementary Table S1 for detailed settings of each simulated scenario.

**IVs selection**	**Method**	**LoS11 (independent pleiotropy)**				**LoS12 (correlated pleiotropy)**			
		$\boldsymbol{\delta_{12} = 0.1}$		$\boldsymbol{\delta_{21} = -0.1}$		$\boldsymbol{\delta_{12} = 0.1}$		$\boldsymbol{\delta_{21} = -0.1}$	
		Mean χ^2 (SD)	Power	Mean χ^2 (SD)	Power	Mean χ^2 (SD)	Power	Mean χ^2 (SD)	Power
N/A	MRCI	54.00 (41.89)	0.98	35.18 (31.46)	0.92	70.72 (51.75)	0.95	63.86 (61.65)	0.88
**exposure-specific true causal SNPs**	MR-Egger	54.50 (18.10)	1.00	49.82 (18.12)	1.00	49.41 (16.98)	1.00	55.72 (17.55)	1.00
	Weighted Mode	25.70 (22.83)	0.71	28.18 (26.64)	0.74	23.55 (23.08)	0.69	23.08 (23.28)	0.64
	MRMix*	N/A	N/A	N/A	N/A	N/A	N/A	N/A	N/A
**significant exposure-associated SNPs excluding potential outcome-associated SNPs**	MR-Egger	3.50 (3.67)	0.33	2.21 (2.30)	0.19	3.34 (4.66)	0.30	5.53 (4.01)	0.56
	Weighted Mode	4.78 (2.88)	0.56	3.78 (2.54)	0.40	3.98 (2.75)	0.47	4.75 (3.24)	0.55
	MRMix	24.15 (16.41)	0.90	21.03 (16.77)	0.88	8.09 (20.19)	0.32	27.40 (17.76)	0.87
**significant exposure-associated SNPs with no exclusion in outcome**	MR-Egger	2.00 (2.90)	0.12	1.47 (2.21)	0.10	0.75 (1.05)	0.01	1.26 (1.67)	0.10
	Weighted Mode	5.74 (3.34)	0.65	4.71 (3.09)	0.54	7.56 (4.21)	0.85	15.59 (7.40)	0.94
	MRMix	22.86 (12.96)	0.97	21.20 (10.95)	0.98	18.57 (15.78)	0.67	35.21 (23.04)	0.78

(Note: *Not applied to MRMix due to its mixture-model assumption.)

Supplementary Table 8. Summary of the reciprocal causal estimates under high polygenicity scenarios. Our method shows nearly unbiased estimates, well-controlled Type I error rate, and adequate power. In the simulation, $\pi_1 = \pi_2 = \pi_c = 1\times10^{-3}$ and $\rho_{C1,C2} = 0.1$. "Empirical SD" is the standard deviation of causal estimates from 100 simulations; "Mean SandwichSE" is the mean value of the standard errors calculated by the Sandwich estimator from 100 simulations. The column 'simID' gives the ID of each simulated scenario, and the parameter settings for each scenario can be found in Supplementary Table S1.

simID	Parameter	True value	Mean estimate	Empirical SD	Mean SandwichSE	Mean χ^2 (SD)	Type I error rate	Power
HiS1	δ_{12}	0.00E+00	-7.65E-03	1.91E-02	2.19E-02	1.09 (1.56)	0.09	N/A
	δ_{21}	0.00E+00	-4.76E-03	1.81E-02	2.18E-02	0.88 (1.27)	0.06	N/A
HiS2	δ_{12}	1.00E-01	8.78E-02	2.17E-02	2.17E-02	22.94 (15.34)	N/A	0.95
	δ_{21}	0.00E+00	-1.19E-02	2.01E-02	2.33E-02	1.23 (1.53)	0.08	N/A
HiS3	δ_{12}	-1.00E-01	-1.09E-01	2.08E-02	1.96E-02	39.49 (21.91)	N/A	0.98
	δ_{21}	0.00E+00	8.92E-04	2.00E-02	1.78E-02	1.33 (1.69)	0.09	N/A
HiS4	δ_{12}	1.00E-01	9.26E-02	1.89E-02	2.09E-02	27.86 (16.59)	N/A	0.96
	δ_{21}	5.00E-02	4.87E-02	1.69E-02	2.11E-02	8.14 (6.15)	N/A	0.68
HiS5	δ_{12}	-1.00E-01	-1.10E-01	2.10E-02	1.96E-02	41.44 (21.45)	N/A	0.99
	δ_{21}	-5.00E-02	-5.81E-02	1.75E-02	1.95E-02	12.66 (9.95)	N/A	0.85
HiS6	δ_{12}	1.00E-01	8.29E-02	1.52E-02	1.94E-02	24.11 (13.38)	N/A	0.97
	δ_{21}	-1.00E-01	-1.14E-01	1.94E-02	2.09E-02	39.14 (20.07)	N/A	0.97
HiS7	δ_{12}	1.00E-01	1.09E-01	2.51E-02	2.56E-02	21.19 (13.2)	N/A	0.99
	δ_{21}	5.00E-02	5.03E-02	2.92E-02	2.38E-02	6.85 (6.84)	N/A	0.52
HiS8	δ_{12}	1.00E-01	1.11E-01	2.57E-02	2.57E-02	23.59 (14.1)	N/A	0.99
	δ_{21}	0.00E+00	8.34E-04	2.38E-02	2.25E-02	1.17 (1.27)	0.07	N/A

Supplementary Table 9. Comparison of genetic correlations (r_g) estimated by MRCI and LDSC under various simulated scenarios. In the table, 'Mean' represents the mean estimates from 100 simulations; 'SD' represents the standard deviation of the 100 estimates. The column 'simID' gives the ID of each simulated scenario, and the parameter settings for each scenario can be found in Supplementary Table S1.

Group	simID	True r_g	LDSC		MRCI	
			Mean	SD	Mean	SD
Low Polygenicity	LoS1	0.000	0.001	0.046	0.001	0.034
($s_{1,2,C}$ model)	LoS2	0.250	0.245	0.082	0.209	0.046
	LoS3	0.100	0.114	0.054	0.108	0.035
	LoS4	0.348	0.297	0.051	0.285	0.037
	LoS5	-0.100	-0.094	0.048	-0.098	0.037
	LoS6	0.149	0.115	0.045	0.112	0.034
	LoS7	0.149	0.147	0.041	0.151	0.034
	LoS8	0.399	0.352	0.050	0.343	0.039
	LoS9	-0.149	-0.150	0.051	-0.150	0.036
	LoS10	0.101	0.083	0.060	0.045	0.036
	LoS11	0.000	-0.002	0.056	0.002	0.040
	LoS12	0.245	0.209	0.070	0.190	0.046
High Polygenicity	HiS1	0.250	0.246	0.034	0.224	0.040
($s_{1,2,C}$ model)	HiS2	0.348	0.344	0.029	0.340	0.037
	HiS3	0.149	0.156	0.032	0.125	0.032
	HiS4	0.399	0.392	0.027	0.411	0.043
	HiS5	0.101	0.118	0.034	0.087	0.028
	HiS6	0.245	0.251	0.033	0.232	0.029
High Polygenicity	$s_{1,2,C}$ (null)	0.250	0.246	0.034	0.224	0.039
(sub-models)	$s_{2,c}$ (null)	0.500	0.498	0.042	0.476	0.036
	$s_{1,2}$ (null)	0.000	0.002	0.033	0.005	0.037
	s_c (null)	0.289	0.295	0.057	0.280	0.051
	$s_{1,2,C}$ (uni-directional)	0.348	0.344	0.029	0.340	0.037
	$s_{2,c}$ (uni-directional)	0.686	0.631	0.041	0.630	0.045
	$s_{1,2}$ (uni-directional)	0.100	0.102	0.030	0.104	0.025
	s_c (uni-directional)	0.402	0.395	0.045	0.336	0.046
	$s_{1,2,C}$ (bi-directional)	0.399	0.392	0.027	0.411	0.042
	$s_{2,c}$ (bi-directional)	0.713	0.649	0.044	0.646	0.043
	$s_{1,2}$ (bi-directional)	0.149	0.150	0.033	0.151	0.028
	s_c (bi-directional)	0.446	0.427	0.045	0.407	0.045

Supplementary Table 10. Estimation with different sample sizes in high polygenicity scenarios. In the table, 'Mean estimate' represents the mean estimates from 100 simulations; 'Empirical SD' represents the standard deviation of the 100 estimates; 'Mean SandwichSE' represents the mean value of the standard error of the parameter using the Sandwich estimator. As sample sizes of GWAS increase, the accuracy becomes higher and standard error becomes smaller. The standard errors estimated by Sandwich estimator are close to the empirical SD in all scenarios, indicating the accuracy of Sandwich estimator. In the simulations, the mixing proportions were set as $\pi_1 = \pi_2 = \pi_c = 1\times10^{-3}$; the heritabilities contributed by Y_1-specific, Y_2-specific and pleiotropic SNPs were set as 0.3, 0.3 and 0.1 respectively, and $\rho_{C1,C2} = 0.1$. (#, samples were 100% overlapped; *, the 20K individuals were completely included in the 50K individuals.)

Parameter	True value	Sample sizes (Y1 / Y2)								
		20K / 20K #			50K / 20K *			50K / 50K #		
		Mean Estimate	Mean SandwichSE	Empirical SD	Mean Estimate	Mean SandwichSE	Empirical SD	Mean Estimate	Mean SandwichSE	Empirical SD
δ_{12}	0.100	0.087	0.056	0.043	0.091	0.027	0.027	0.093	0.021	0.019
δ_{21}	0.050	0.055	0.053	0.040	0.036	0.068	0.046	0.049	0.021	0.017
