# Supplementary material for: Reciprocal causation mixture model for robust Mendelian randomization analysis using genome-scale summary data
Source: Nat Commun. 2023 Feb 28;14:1131. doi: 10.1038/s41467-023-36490-4 (PMC9975185; doi:10.1038/s41467-023-36490-4)
Supplement: Supplementary file 3 — Editorial Assessment Report [file 41467_2023_36490_MOESM3_ESM.pdf]

nature portfolio

GUIDED OPEN ACCESS

nature genetics | nature communications | communications biology

Contents of this report

- **Manuscript details**: overview of your manuscript and the editorial team.
- **Review synthesis**: summary of the reviewer reports provided by the editors.
- **Editorial recommendation**: personalized evaluation and recommendation from all 3 journals.
- **Annotated reviewer comments**: the referee reports with comments from the editors.
- **Open research evaluation**: advice for adhering to best reproducibility practices.

About the editorial process

Because you selected the **Nature Portfolio Guided Open Access option**, your manuscript was assessed for suitability in three of our titles publishing high-quality work across the spectrum of genetics research: *Nature Genetics, Nature Communications*, and *Communications Biology*. More information about Guided Open Access can be found here.

Collaborative editorial assessment

Your editorial team discussed the manuscript to determine its suitability for the Nature Portfolio Guided OA pilot. Our assessment of your manuscript takes into account several factors, including whether the work meets the **technical standard** of the Nature Portfolio and whether the findings are of **immediate significance** to the readership of at least one of the participating journals in the Nature Portfolio Guided Open Access genetics cluster.

Peer review

Experts were asked to evaluate the following aspects of your manuscript:

- **Novelty** in comparison to prior publications;
- **Likely audience** of researchers in terms of broad fields of study and size;
- **Potential impact** of the study on the immediate or wider research field;
- **Evidence** for the claims and whether additional experiments or analyses could feasibly strengthen the evidence;
- **Methodological detail** and whether the manuscript is reproducible as written;
- Appropriateness of the literature review.

Editorial evaluation of reviews

Your editorial team discussed the potential suitability of your manuscript for each of the participating journals. They then discussed the revisions necessary in order for the work to be published, keeping each journal's specific editorial criteria in mind.

Journals in the Nature portfolio will support authors wishing to transfer their reviews and (where reviewers agree) the reviewers' identities to journals outside of Springer Nature.

If you have any questions about review portability, please contact our editorial office at guidedoa@nature.com.

nature portfolio

GUIDED OPEN ACCESS
nature genetics nature communications communications biology

Manuscript details

Tracking number	Submission date	Decision date
GUIDEDOA-21-00193	15 July 2021	5 October 2021

**Title**	Reciprocal causation mixture model for robust mendelian randomization analysis using genome-scale summary data	**Corresponding author**	Pak Sham<br>**Affiliation:** The University of Hong Kong
**Preprint information**	There is a preprint of this manuscript posted at **[link]**	**Peer review type**	Single-blind

Editorial assessment team

**Primary editor**	**Wei Li**<br>**Home Journal:** ***Nature Genetics*****, ORCID:** 0000-0002-7885-1775<br>**Email:** wei.li@nature.com
**Editorial team members**	**Margot Brandt**, *Nature Communications*, ORCID: 0000-0002-9434-794X<br>**George Inglis**, *Communications Biology*, ORCID: 0000-0002-9069-5242
**About your primary editor**	Wei Li obtained her Ph.D. in Genetics and Genomics from Shanghai Institutes for Biological Sciences, Chinese Academy of Sciences in Bin Han's plant genomics and multi-omics research group. For her postdoctoral work, Wei moved to The Rockefeller University and then Weill Cornell Medical College of Cornell University at Anthony A. Sauve's laboratory to study signaling networks in mammalian metabolic diseases such as diabetes. She joined the *Nature Genetics* team in 2017.

Editorial assessment and review synthesis

**Editor's summary and assessment**	The authors presented a robust method, MRCI (Mixture model Reciprocal Causation Inference), for reciprocal causal inference between pairs of phenotypes simultaneously using the genome-scale summary statistics of the two phenotypes and reference linkage disequilibrium (LD) information. Compared with existing MR approaches, MRCI tested for reciprocal causation without the selection of instrumental variables (IVs), thus making full use of genetic information while explicitly modelling pleiotropy. Results from extensive simulation studies demonstrated the robustness of this method in a wide range of scenarios. When applied to real GWAS summary data on 3 common diseases (type 2 diabetes, ischemic stroke, and coronary heart disease) and 16 putative risk factors, the method inferred causal effects consistent with current knowledge, including a reciprocal causal relationship between type 2 diabetes and body mass index.<br><br>While the editors jointly decided to send this manuscript out to review, we recognized that there were many existing MR methods. We appreciate the features of testing for reciprocal causation without the selection of IVs and inferring the causal paths simultaneously in both directions, though have some concerns about the technical advance of the MRCI method as well as the biological insights obtained from applications to real GWAS data.
**Editorial synthesis of reviews**	For consideration at *Nature Communications*, we ask that all specific points by the reviewers are addressed in the revision, with particular emphasis on extending the simulations to address reviewer 1's point 1, comparing to other methods as suggested by reviewers 1 and 2 and more thoroughly describing the method and simulations, as requested by reviewer 3.<br><br>*Communications Biology* would similarly request that a revised manuscript compare to other approaches and elaborate on the Methods and simulations, but would not require additional simulations to be included in the Results.

Editorial recommendation

nature genetics	Revision not invited	The degree of technical or conceptual advance has not matched the criteria for further consideration at *Nature Genetics*.
nature communications	Minor Revisions	While there are several methods addressing the limitations of Mendelian randomization, we agree with the reviewers that this manuscript is a valuable contribution, and we would consider publishing it if the simulations could be extended and the advantages of the current method were made clear.
communications biology	Minor Revisions	In light of the reviewers' positive feedback, *Communications Biology* would be interested in a revised manuscript that better describes limitations of the method and textually distinguishes it from similar, alternative approaches.

Next steps

Recommendation Summary

- Option 1: Revise for consideration at *Nature Communications.*
- Option 2: Revise for consideration at *Communications Biology*.

See the previous page for details. ***Nature Genetics*** *can no longer consider the manuscript because the degree of technical or conceptual advance has not matched the journal's criteria. If you choose to submit the manuscript via Guided Open Access, only Nature Communications and Communications Biology will discuss the revision.*

Revision

To follow our recommendation, please upload the revised manuscript, along with your point-by-point response to the reviewers' reports and editorial advice **using the link provided in the decision letter**. Should you need assistance with our manuscript tracking system, please contact Adam Lipkin, our Nature Portfolio Guided OA support specialist, at guidedOA@nature.com.

Revision checklist

- Cover letter, stating to which journal you are submitting
- Revised manuscript
- Point-by-point response to reviews
- Updated **Reporting Summary** and **Editorial Policy Checklist**
- Supplementary materials (if applicable)

Submission elsewhere

To a journal outside of Nature Portfolio

If you choose to submit your revised manuscript to a journal at another publisher, we can share the reviews with another journal outside of the Nature Portfolio if requested. You will need to request that the receiving journal office contacts us at guidedOA@nature.com. We have included editorial guidance below in the reviewer reports and open research evaluation to aid in revising the manuscript for publication elsewhere.

Annotated reviewer reports

The editors have included some additional comments on specific points raised by the reviewers below, to clarify requirements for publication in the recommended journal(s). However, please note that all points should be addressed in a revision, even if an editor has not specifically commented on them.

Reviewer #1

**Reviewer #1**	This reviewer has not chosen to waive anonymity. The reviewer's identity can only be shared with representatives of an established journal editorial office.
**Reviewer #1 expertise**<br>Summarised by the editor	statistical genetics, epidemiology
**Editor's comments about this review**	This reviewer has major concerns regarding the simulations, which should be thoroughly addressed for further consideration. This reviewer also points out other aspects of the analyses and presentation that need to be improved or clarified.

Reviewer #1 comments

**Overview**	**Overall significance**<br>Liu et al propose a normal mixture model for the genetic effects on two traits and include bidirectional causal effects that can be estimated under Mendelian randomisation assumptions allowing for both correlated and uncorrelated pleiotropy. The method is shown to work well under simulations and improve upon standard and related methods including CAUSE and MRMIX. Although the authors are not the first to propose this type of model, there are some very nice innovations in this work, including the composite likelihood with sandwich variance to allow for LD, and the model averaging to allow for missing components.<br>A very similar method has been on medRxiv for a couple of years now, the most recent version here https://www.medrxiv.org/content/10.1101/2020.01.27.20018929v2.full. I would not demand discussion of a work that has yet to complete peer review, but the authors should be honest if they have drawn upon this related work at all.<br>**Impact**<br>Altogether this looks like a substantive and useful contribution.

Specific comments		
**#**	**Reviewer comment**	**Editorial comment**
**1**	**Strength of the claims**<br>1. The authors performed a lot of simulations and I am not one to quibble over details. However there are a few overarching issues.<br>a. Only the one-sample design was simulated. This creates weak instrument bias towards the observational association, which was observed in the simulations, but in the two-sample design the bias is towards the null. Given the popularity of the two-sample design, there should be some simulations comparing MRCI to standard methods in this case.<br>b. Furthermore, since the model allows partial sample overlap, there should be some simulations showing that the method works correctly in this situation.<br>c. Similarly, since the model allows for population stratification, there should also be some corresponding simulations.<br>d. The authors observed increased bias when IVs are selected according to sample associations with exposure. This looks like a winner's curse effect, which is a different problem to pleiotropy and weak instrument bias. Since this is often done in practice, the authors are welcome to demonstrate the effect, but it would also be helpful to see what happens when IVs are selected from an independent sample, as done eg in Zhao Q et al Int J Epidemiol 2019. Although the present results using "true" IVs get at this somewhat, it would still help if the authors clarified the presence of winner's curse effect.<br>e. In the time-honoured way, the authors' method performed better than its competitors and recovered the desired statistical properties. When would it do less well? What assumptions does it require, that other methods may not? In particular, why does MRCI do better than CAUSE and MRMIX, when the models are so similar? What is creating the	**The extension of simulations to a two-sample design and demonstration of robustness to sample overlap are important revisions for *Nature Communications*.**<br><br>**While *Communications Biology* would strongly encourage you to include this simulation, at a minimum the reliance on a one-sample design should be discussed as a limitation.**

	improvement? Why does CAUSE have increased type-1 error? Insights are welcome.	
2	2. Page 6, "estimation assuming the full model often did not correctly infer the absent component". I wondered whether this might be as result of the numerical maximisation when the mixing proportions must be within [0,1]. Optimisation can work better when such variables are parameterised on the logit scale and I wondered whether this was done. Similar comments apply to the variances.	
3	3. In the data examples, some of the inferred effects are implausible, such as T2D on birth weight. Also CAD on BMI and T2D on BMI are doubtful: although indeed these diseases can lead to weight loss, what the data show is that genetic predisposition to these diseases is negatively correlated with BMI in the general population (because the BMI effects are taken from population studies). And because most people in the BMI studies do not have CAD or T2D, we are probably not seeing causal effects of the diseases themselves. What aspects of this model are leading to implausible inferences?	
4	4. As seen in figure 1, the model is perfectly symmetric. This suggests that there are (at least) two solutions that fit any data set equally well. How do we deal with this?	
5	5. The sandwich variance is a very nice idea, but it does require a large sample. Is it possible to give some guidance on the minimal sample size where this approach is reasonable?	
6	6. Page 13, unclear how the case/control data were simulated. "45,000 individuals to simulate a case-control study with disease prevalence 0.05 and case:control ratio 1:2", does this mean there were 0.05*45k=2,250 cases and twice as many (4500) controls? Or does it mean 15,000 cases and 30,000 controls, in which case a much larger number of individuals must be simulated to obtain that many cases.	
7	7. Please check referencing, eg ref 26 is missing authors and publisher, ref 28 volume and page numbers.	

**8**	8. Supp text, top page 5, should BIC read AIC?	
**9**	9. Could the authors please make their software available?	**Please note that making the code and software available would be required prior to acceptance in either *Nature Communications* or *Communications Biology*.**

**Reviewer #2**	
**Reviewer #2**	This reviewer has not chosen to waive anonymity. The reviewer's identity can only be shared with representatives of an established journal editorial office.
**Reviewer #2 expertise**<br>Summarised by the editor	statistical genetics, epidemiology
**Editor's comments about this review**	This reviewer has provided an overall positive assessment of the study, but requests further justification using empirical datasets

**Reviewer #2 comments**	
**Overview**	**Overall significance**<br>The proposed approach MRCI mainly focused on handling potentially correlated horizontal pleiotropy which is an essential but challenging task in Mendelian randomization. It is an interesting and solid work in general. The authors provided a methodologically robust framework and demonstrated its utility by simulations assuming strongly correlated pleiotropy effects. However, as the authors pointed out, the hypothesis of a strongly correlated pleiotropy effect needs to be justified in the further with evidence from empirical datasets. The feature of the "two-way test in one run" is interesting but a bit trivial since it won't take much time to run regular MR on the other way when the GWAS datasets have been harmonized.<br>**Impact**<br>Included above.

Specific comments		
**#**	**Reviewer comment**	**Editorial comment**
**1**	**Strength of the claims**<br>**Non-technical comments:**<br>1. It is hard to write comments without line numbers and page numbers.<br>2. Please include figures and figure legends in the main text during peer-review. Otherwise, the reviewers need to jump back and force three different places which is annoying.<br>2. 3. The authors may also notice the MR approach "MRCIP" published very recently. Both "MRCI" and "MRCIP" target on correlated pleiotropy effect. What a coincidence. I suggest the authors find another name since people may be confused about them (e.g., consider MRCIP as an extension of MRCI).	**For the figures and figure legends, we would leave this point up to the authors' discretion.**
**2**	**Technical comments:**<br>1. The second paragraph of the introduction needs to be reorganized. The summary of the MR history is incomplete.<br>The logic is not clear by saying "However, using multiple SNPs as IVs also increases the chance of horizontal pleiotropy for some SNPs" given the context that they just mentioned "weighted median" and "weighted mode".<br>The authors highlighted CAUSE as a recent advance after the sentence "… they still involve the selection of independent IVs, which may exclude the majority of SNPs". However, CAUSE uses pruned/independent IVs.<br>From my understanding, the point to develop MRCI is providing a robust solution when the proportion of IVs with pleiotropy effect is much higher than its setting in CAUSE. CAUSE already provided a way to address correlated pleiotropy. The authors need to highlight it in the background if they agree with this point.	
**3**	2. The authors need to give and clear definition for what does "correlated pleiotropy" mean in the main text. It probably means correlated gamma_c1 and gamma_c2 in	

	Figure 1. However, in the MR field, people are more familiar with the idea of the "violation of the InSIDE assumption". I think the "correlated pleiotropy" and "violation of the InSIDE" are consistent when considering the Gc (Figure 1) as heritable Unobserved confounding factors. Readers will appreciate it if the authors could use established concepts.	
4	3. In the first paragraph of "Estimation and hypothesis testing under the full model", I don't agree with the claim "However, IV-based MR methods generated biased estimates when GWAS significant SNPs for the exposure phenotype were used as IVs, especially under scenarios with correlated pleiotropy" based on the performance of "weighted mode".	
5	4. Same paragraph as mentioned above, I think it is good to include CAUSE as a player in Figure 2. Although the authors made the comparison between CAUSE and MRCI in the following section.	
6	5. In the second paragraph of "Estimation and hypothesis testing under the full model", please provide more details about "simulations with correlated pleiotropy" here.	
7	6. Restrictions need to be mentioned in the first sentence of the second paragraph of "Estimation and hypothesis testing under the full model". e.g., by assuming extensive correlated pleiotropy effect.	
8	7. Sections "Estimation and hypothesis testing under sub-models" and "Comparison with CAUSE and MRMix". Although I can certainly see the flexibility of the approach from a methodological perspective (which is great), it is very hard for me to picture a real scenario that trait-specific SNPs are absent for one or both phenotypes. Discusses about the relationship between such extreme conditions and the observations of genetic correlations from empirical data is needed.	
9	8. The observation of a reciprocal causal relationship between BMI and T2D is interesting. I am trying to get my head around these biologically reciprocally correlated phenotypes. I am wondering whether using a GWAS of	

	T2D_adj_BMI and a GWAS of BMI would give us a more intuitive picture of the causality from T2D to BMI.	
**10**	9. Another two similar approaches have been published online recently (listed below). Comments in the Discussion will help readers to find their best fit.<br>Xu, Siqi, Wing Kam Fung, and Zhonghua Liu. "MRCIP: a robust Mendelian randomization method accounting for correlated and idiosyncratic pleiotropy." Briefings in Bioinformatics (2021).<br>Cheng, Qing, Tingting Qiu, Xiaoran Chai, Baoluo Sun, Yingcun Xia, Xingjie Shi, and Jin Liu. "MR-Corr2: a two-sample Mendelian randomization method that accounts for correlated horizontal pleiotropy using correlated instrumental variants." Bioinformatics (2021).<br>**Reproducibility**<br>**Included above.**	**Since other similar methods exist, we encourage authors to follow the reviewer's suggestion to highlight the advantages of the present method and in which situations it would be most appropriate.**<br><br>**This point would also be required for *Communications Biology*.**

**Reviewer #3**	
**Reviewer #3**	This reviewer has not chosen to waive anonymity. The reviewer's identity can only be shared with representatives of an established journal editorial office.
**Reviewer #3 expertise**<br>Summarised by the editor	Mendelian randomization, epidemiology
**Editor's comments about this review**	This reviewer points out a few limitations of the method and provides suggestions for improvement. This reviewer also highlights the limited reproducibility, which should be improved for further consideration.
**Reviewer #3 comments**	
**Overview**	**Overall significance**<br>This paper presents a novel MR estimation method that claims to make use of genome-wide associations and SNPs in LD to estimate bi-directional causal effects. This method is potentially interesting and its links to existing methods discussed appropriately. However, not enough description of the simulations or application

	are given to fully assess the method.<br>**Impact**<br>This paper provides a novel method that could influence and improve the methods available for pleiotropy robust MR analysis however the limitations of the method, scenarios in which it would be most relevant and implementation of the method need to be more fully explored for its benefit to the field to be fully achieved.

Specific comments

#	Reviewer comment	Editorial comment
**1**	**Strength of the claims**<br>1. This paper claims to use genome-wide SNPs in the estimation however in the simulation SNPs that have no association with either phenotype appear to be excluded from the analysis. Is this also the case in the applied analysis? In this case what rule is used to determine whether or not SNPs should be included in the applied analysis?	
**2**	2. From the description of the distribution of the SNP effects, it appears all of the pleiotropic effects are assumed to be balanced. Given the potential issues that arise around unbalanced pleiotropy this is an important limitation of the method that needs to be highlighted clearly. This is particularly true around the discussion of correlated pleiotropy which is often considered to mean unbalanced directional pleiotropy in the same direction on each exposure. A discussion of this limitation and ideally a simulation illustrating the effect of unbalanced pleiotropy on the method would strengthen the manuscript.	**While we would encourage you to also include a simulation, at a minimum this point must be discussed as a limitation for consideration at *Communications Biology*.**
**3**	3. Correlated pleiotropy is potentially a significant problem for MR analysis so it is really good that the authors consider this in their simulations, however have they considered a higher level of correlated pleiotropy? How sensitive is the method to higher levels of correlated pleiotropy?	
**4**	4. Have you considered how sensitive the estimation method is to differences in instrument strength or the level of pleiotropy associated with the SNPs for each	

	exposure? It would be beneficial to discuss these limitations and simulate the effect of (particularly) different levels of pleiotropy on the different phenotypes.	
**5**	5. In the section on sub-models and model averaging; can the authors expand on how using the submodels makes their estimation robust and what it is robust to?	
**6**	6. In the introduction I was confused by the introduction of MR CAUSE as a model that uses LD pruned SNPs immediately after the discussion of the benefits of using non-independent SNPs since the LD pruning required by MR CAUSE restricts the analysis to independent SNPs.	
**7**	**Reproducibility**<br>Although the analysis appears to be appropriate (given the caveats of the limitations that haven't been explored discussed above), neither the simulations or the applied analysis are sufficiently described for reproducibility or full assessment of the method.<br>1. I didn't understand how the data for the simulation had been generated. The paper suggests that it has been selected from UK Biobank, but in that case what is simulated? The description needs to be expanded on to fully understand how the simulations have been conducted.<br>2. There is very limited description of the data used in the applied analysis. More than just a reference is needed given that this data is being used to illustrate a novel method.	**For *Nature Communications*, a thorough description of the method and simulations is required.**<br><br>**This point would also be required by *Communications Biology*, for the sake of reproducibility. Please also refer to the Open Research Evaluation.**

Open research evaluation

Data availability

Data availability statement

Please add a Data Availability statement. Please ensure that your Data Availability statement includes accession details for deposited data, mentions where Source data can be found, and states that all other data are available from the corresponding author (or other sources, as applicable) on reasonable request.

More information about our data availability policy can be found here: https://www.nature.com/nature-portfolio/editorial-policies/reporting-standards#availability-of-data

See here for more information about formatting your Data Availability Statement: http://www.springernature.com/gp/authors/research-data-policy/data-availability-statements/12330880

Mandatory data deposition

Data availability: This journal strongly supports public availability of data and custom code associated with the paper in a persistent repository where they can be freely and enduringly accessed or as a supplementary data file when no appropriate repository is available. If data and code can only be shared on request, please explain why in your data Availability Statement, and also in the correspondence with your editor. For more information, please refer to https://www.nature.com/nature-research/editorial-policies/reporting-standards#availability-of-data

Please ensure that datasets deposited in public repositories are now publicly accessible, and that accession codes or DOI are provided in the "Data Availability" section. As long as these datasets are not public, we cannot proceed with the acceptance of your paper. For data that have been obtained from publicly available sources, please provide a URL and the specific data product name in the data availability statement. Data with a DOI should be further cited in the methods reference section.

Code Availability: Custom code should be deposited in a DOI-minting repository such as Zenodo, Gigantum or Code Ocean and cited in the reference list. Full details about how the code can be

accessed and any restrictions must be described in the Code Availability statement. See Nature policy here: (https://www.nature.com/nature-research/editorial-policies/reporting-standards#availability-of-computer-code).

Reporting & reproducibility

Nature Portfolio journals allow unlimited space for Methods. The Methods must contain sufficient detail such that the work could be repeated. It is preferable that all key methods be included in the main manuscript, rather than in the Supplementary Information. Please avoid use of “as described previously” or similar, and instead detail the specific methods used with appropriate attribution.

Reproducibility: Please state in the legends how many times each experiment was repeated independently with similar results. This is needed for all experiments, but is particularly important wherever results from representative experiments (such as micrographs) are shown. If space in the legends is limiting, this information can be included in a section titled “Statistics and Reproducibility” in the methods section.

Statistics and data presentation

When choosing a color scheme please consider how it will display in black and white (if printed), and to users with color blindness. Please consider distinguishing data series using line patterns rather than colors, or using optimized color palettes such as those found at https://www.nature.com/articles/nmeth.1618

The use of colored axes and labels should be avoided. Please avoid the use of red/green color contrasts, as these may be difficult to interpret for colorblind readers.

Data presentation: Please ensure that data presented in a plot, chart or other visual representation format shows data distribution clearly (e.g. dot plots, box-and-whisker plots). When using bar charts, please overlay the corresponding data points (as dot plots) whenever possible and always for n ≤ 10. (Please see the following editorial for the rationale behind this request and an example https://www.nature.com/articles/s41551-017-0079).

Statistics: Wherever statistics have been derived (e.g. error bars, box plots, statistical significance) the legend needs to provide and define the n number (i.e. the sample size used to derive statistics) as a precise value (not a range), using the wording "n=X biologically independent samples/animals/cells/independent experiments/n= X cells examined over Y independent experiments" etc. as applicable.

Legends requiring revision:

1. Please note that this information is missing in the legends of supplementary figures 3a-b.

Please note that statistics such as error bars significance and p values cannot be derived from n<3 and must be removed in all such cases.

We strongly discourage deriving statistics from technical replicates, unless there is a clear scientific justification for why providing this information is important. Conflating technical and biological variability, e.g., by pooling technically replicates samples across independent experiments is strongly discouraged. (For examples of expected description of statistics in figure legends, please see the following https://www.nature.com/articles/s41467-019-11636-5 or https://www.nature.com/articles/s41467-019-11510-4).

All error bars need to be defined in the legends (e.g. SD, SEM) together with a measure of centre (e.g. mean, median). For example, the legends should state something along the lines of "Data are presented as mean values +/- SEM" as appropriate.

All box plots need to be defined in the legends in terms of minima, maxima, centre, bounds of box and whiskers and percentile.

Legends requiring revision:

1. Please note that the box plots need to be defined in terms of minima, maxima, centre, bounds of box and whiskers and percentile in the legend of supplementary figure 3b.

The figure legends must indicate the statistical test used. Where appropriate, please indicate in the figure legends whether the statistical tests were one-sided or two-sided and whether adjustments were made for multiple comparisons.

For null hypothesis testing, please indicate the test statistic (e.g. F, t, r) with confidence intervals, effect sizes, degrees of freedom and P values noted.

Please provide the test results (e.g. P values) as exact values whenever possible and with confidence intervals noted.

Legends requiring revision:

1. Please indicate the statistical test used for data analysis and where appropriate, please specify whether it was one-sided or two-sided and whether adjustments were made for multiple comparisons, in the legends of figures 2d, 3b, 4b, 5.

Other notes

We have included as an attachment to the decision letter a version of your Reporting Summary with a few notes. This is mainly for your information, but we hope it is helpful when preparing your revised manuscript. If you decide to resubmit the manuscript for further consideration, please be sure to include an updated Reporting Summary.
